# The aminosterol Claramine inhibits β-secretase 1–mediated insulin receptor cleavage

**DOI:** 10.1016/j.jbc.2021.100818

**Published:** 2021-05-23

**Authors:** Bénédicte Gaborit, Roland Govers, Alexandre Altié, Jean Michel Brunel, Pierre Morange, Franck Peiretti

**Affiliations:** 1 INSERM, INRAE, C2VN, Aix Marseille University, Marseille, France; 2 Endocrinology, Metabolic Diseases and Nutrition Department, APHM, Marseille, France; 3 INSERM, SSA, MCT, Aix Marseille University, Marseille, France; 4 Hematology Laboratory, La Timone Hospital, APHM, Marseille, France

**Keywords:** insulin receptor, beta-secretase 1 (BACE1), liver, diabetes, *O*-GlcNacylation, proteasome, lysosome, autophagy, AD, Alzheimer disease, APP, amyloid precursor protein, BACE1, β-secretase 1, BAF, Bafilomycin A1, ER, endoplasmic reticulum, ERAD, ER-associated degradation, ERLAD, ER-to-lysosome-associated degradation, ESP, early secretory pathway, HBP, hexosamine biosynthetic pathway, HFD, high-fat diet, IR, insulin receptor, PC, proprotein convertase, PTM, posttranslational modification, TGN, *trans* Golgi network

## Abstract

The cleavage of the insulin receptor by β-secretase 1 (BACE1) in the liver increases during diabetes, which contributes to reduce insulin receptor levels and impair insulin signaling. However, the precise signaling events that lead to this increased cleavage are unclear. We showed that BACE1 cleaves the insulin receptor in the early secretory pathway. Indeed, coimmunoprecipitation experiments reveal the interaction of the proforms of the two proteins. Moreover, fragments of insulin receptor are detected in the early secretory pathway and a mutated form of BACE1 that retains its prodomain cleaves an early secretory pathway-resident form of the insulin receptor. We showed that BACE1 proform levels are regulated by proteasome and/or lysosome-dependent degradation systems whose efficiencies are dependent on the *O*-GlcNacylation process. Our results showed that enhanced *O*-GlcNacylation reduces the efficiency of intracellular protein degradation systems, leading to the accumulation of the proform of BACE1 in the early secretory pathway where it cleaves the precursor of the insulin receptor. All these dysregulations are found in the livers of diabetic mice. In addition, we performed a screen of molecules according to their ability to increase levels of the insulin receptor at the surface of BACE1-overexpressing cells. This approach identified the aminosterol Claramine, which accelerated intracellular trafficking of the proform of BACE1 and increased autophagy. Both of these effects likely contribute to the reduced amount of the proform of BACE1 in the early secretory pathway, thereby reducing insulin receptor cleavage. These newly described properties of Claramine are consistent with its insulin sensitizing effect.

The insulin receptor (IR) mediates the metabolic and mitogenic effects of insulin. Dysfunctions in insulin signaling are responsible for the metabolic syndrome and type 2 diabetes (1) and are also implicated in a variety of cancers (2, 3) and increasingly associated with neurodevelopmental disorders (4, 5).

The decreased IR tyrosine kinase activity with reduction in post-receptor signaling is one of the causes of insulin signaling dysfunction. This aspect has been studied relentlessly for decades, and progress in this area is regularly and comprehensively reviewed (6). IR content reduction has long been suspected to contribute to the defective insulin signaling and diabetes progression. This has been firmly proven by showing that restoring IR expression in the liver of adult diabetic mouse models improves the diabetic phenotypes (7). Moreover, studies have demonstrated that mechanisms regulating density of cell surface functional IR have significant effects on insulin cell signaling. In this line, it was reported that E3 ubiquitin ligases MARCH1 in liver (8) or mitsugumin 53 in muscle (9) impaired insulin action by targeting cell surface IR for ubiquitin-dependent degradation, resulting in a reduction of its cell surface density. In liver, IR cell surface internalization, controlled by the module p31^comet^-MAD2-BUBR1, was shown to be a mechanism regulating insulin signaling (10). In adipocytes, redirection of internalized IR from endosomes to the plasma membrane, controlled by the intracellular sorting receptor SORLA, regulates IR cell surface expression and insulin signaling (11). We have previously established that IR ectodomain is cleaved by BACE1 (12). This cleavage occurs in the liver and increases during diabetes, thus reducing the density of functional IR on the cell surface. Of interest, BACE1 inhibition restores functional cell-surface IR and increases insulin signaling, making the liver BACE1-dependent IR cleavage regulation system an original target for counteracting impaired insulin signaling.

The purpose of this study was to elucidate the mechanisms that regulate BACE1-dependent cleavage of IR during diabetes to identify regulatory steps that could be pharmacologically targeted to reduce IR cleavage and improve insulin sensitivity.

## Results

### BACE1-dependent cleavage of IR occurs in the early secretory pathway

Our previous results suggested that BACE1 zymogen (proBACE1, with its prodomain) was responsible for IR cleavage. This possibility was thoroughly investigated below.

**Figure 1:**
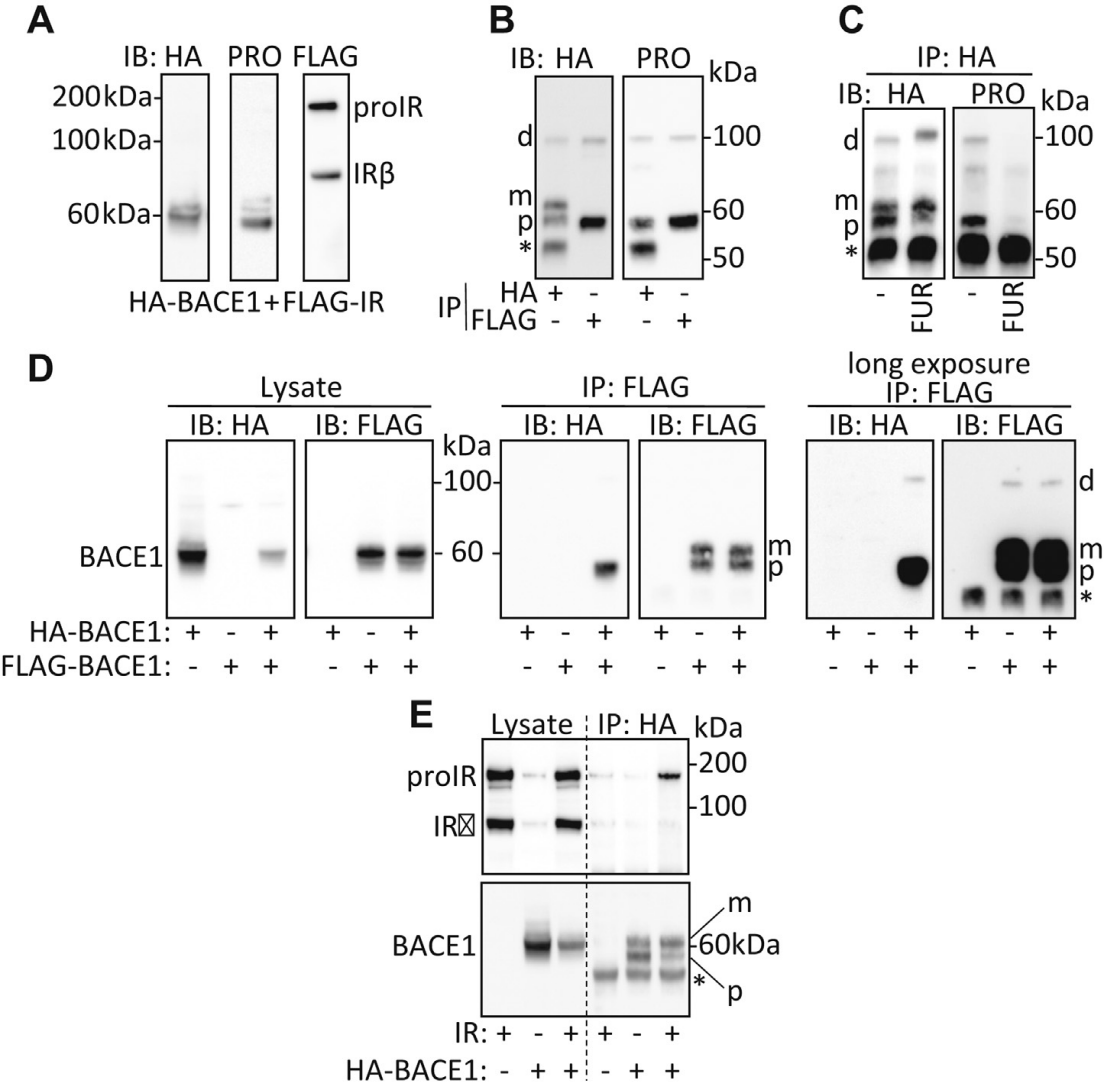
**proBACE1 interacts with proIR.** HA-BACE1 and FLAG-IR overexpressed in HEK293 cells were detected by immunoblot (IB) using anti-HA, anti-BACE1 prodomain (PRO), and anti-FLAG antibodies in (*A*) cell lysates and (*B*) after immunoprecipitation (IP) of HA-BACE1 or FLAG-IR using the corresponding anti-tag antibodies. *C*, HA-BACE1 was immunoprecipitated (IP: HA) from lysates of cells expressing HA-BACE1 alone or with furin (FUR) then BACE1 and proBACE1 were detected. *D*, HA-BACE1 and/or FLAG-BACE1 were expressed as indicated, then BACE1 was detected in cell lysates and after FLAG-BACE1 immunoprecipitation (IP: FLAG); *right panel*: long exposure of the blot. *E*, HA-BACE1 and/or IR were expressed as indicated, then BACE1 and IR were detected in cell lysates and after HA-BACE1 immunoprecipitation. Positions of IR precursor (proIR), BACE1 dimer (d), proBACE1 (p), mature BACE1 (m), and heavy chain immunoglobulin (∗) are indicated.

**Figure 2:**
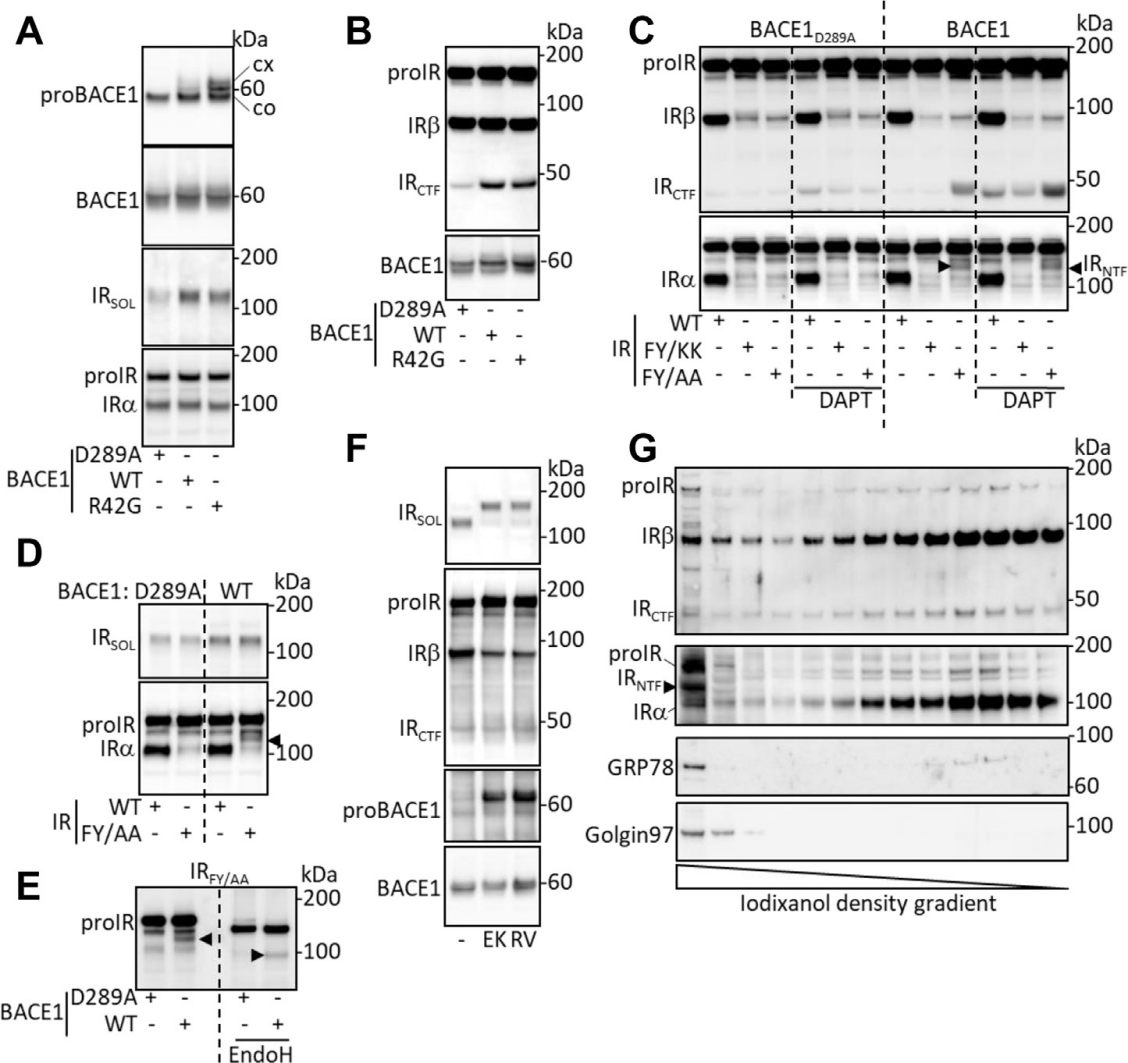
**proBACE1 cleaves proIR in the early secretory pathway.** Wildtype (WT), inactive (D289A), or PC-resistant (R42G) forms of BACE1 were overexpressed with IR or its indicated mutated forms. *A*, proBACE1, total BACE1, and IRα subunit were detected in cell lysates; IR_SOL_ was detected in conditioned media. *B*, cells were treated with DAPT (10 μM, 17 h), then IRβ subunit and BACE1 were detected in cell lysates. *C*, cells were treated with DAPT, then IRβ and IRα subunits were detected in cell lysates. *D*, IRα subunit and IR_SOL_ were detected in cell lysates and in conditioned media, respectively. *E*, cell lysates were treated with EndoH, and IRα subunit was detected. *F*, BACE1 and IR-expressing cells were treated with Dec-RVKR-CMK (RV; 20 μM, 17 h) or transfected with EK4 expression vector (EK) to inhibit PC, then IR_SOL_ was detected in the conditioned media and IRβ subunit, proBACE1, and BACE1 were detected in cell lysates. *G*, distribution of IR, IR_CTF_, and IR_NTF_ in subcellular membrane vesicles of HEPG2 cells fractionated by iodixanol density gradient. Golgi apparatus- and ER-rich fractions are identified by the detection of Golgin 97 and GRP78, respectively. Positions of IR precursor (proIR), IR C-terminal fragment (IR_CTF_), IR N-terminal fragment (IR_NTF_, *black arrowhead*), and core glycosylated (co) and complex N-glycosylated (cx) proBACE1 are indicated.

In lysates of cells expressing FLAG-tagged IR and HA-tagged BACE1, IR precursor (proIR composed of a single polypeptide chain) and mature IR (derived from the cleavage of proIR by furin and yielding the α and β subunits of IR) (Fig. S1) were detected with the antibody specific for the C-terminal region of IR (Fig. 1*A*). proBACE1 was detected by the antibody specific for BACE1 prodomain (below 60 kDa), whereas HA tag antibodies detected a form of BACE1 that migrates slower than proBACE1 (around 60 kDa) that likely corresponds to mature BACE1 (without its prodomain but with its complex glycans moiety) (Fig. 1*A*). Once immunoprecipitated, BACE1 was detected by immunoblot as two major distinct bands (Fig. 1*B*): the lowest one corresponding to proBACE1, since it was also detected by the antibody specific for BACE1 prodomain, and the upper one being the mature BACE1. A 100-kDa HA-tagged protein was also detected. proBACE1 and the 100-kDa HA-tagged protein coimmunoprecipitated with IR and were both detected with BACE1 prodomain-specific antibodies (Fig. 1*B*). Of interest, overexpression of the proprotein convertase (PC) furin, which increases the cleavage of BACE1 propeptide (13), reduced the detection of proBACE1 and that of the 100-kDa protein (Fig. 1*C*) while allowing the detection of a HA-tagged protein that migrated slightly above 100 kDa. These features suggest that the 100-kDa protein is a high-molecular-weight form of proBACE1 and that the protein above 100 kDa, detected upon furin overexpression, is a high-molecular-weight form of mature BACE1. The nature of the 100-kDa proBACE1 was investigated by coimmunoprecipitation of overexpressed HA-tagged BACE1 and FLAG-tagged BACE1. Immunoprecipitation of FLAG-BACE1 pulled down the proform of HA-BACE1 and that of the 100-kDa HA-tagged BACE1 (Fig. 1*D*), suggesting that proBACE1 is expressed in cells in a nonmonomeric form and that the 100-kDa high-molecular-weight form of proBACE1 is a reduction and SDS-resistant dimer of proBACE1. Likewise, the high-molecular-weight form of BACE1 detected upon furin overexpression (Fig. 1*C*) is likely a dimer of mature BACE1. In addition, immunoprecipitation of BACE1 mostly pulled down proIR (Fig. 1*E*). These results support that proIR interacts with monomeric and/or dimeric proBACE1. However, this interaction does not necessarily imply that proBACE1 is responsible for IR cleavage. To address this concern, we generated BACE1_R42G_ that resists PC-induced maturation as shown by the detection of a slow migrating form of proBACE1 (Fig. 2*A*), which likely corresponds to proBACE1 that crossed the Golgi stack and acquired its complex N-glycosylation, as previously demonstrated (14). BACE1_R42G_ and wildtype BACE1 were similarly effective in cleaving IR as shown by the release of soluble IR (IR_SOL_) in the culture media (Fig. 2*A*) and the generation of the transmembrane IR C-terminal fragment (IR_CTF_) (Fig. 2*B*). By comparison, small amounts of IR_SOL_ and IR_CTF_ were generated when the inactive BACE1_D289A_ was overexpressed. IR_CTF_ is normally cleaved by the γ-secretase, which generates an unstable fragment of its intracellular domain (IR_ICD_) that is rapidly degraded by the proteasome; therefore, inhibition of γ-secretase with DAPT is mandatory to observe an IR_CTF_ that best reflects the BACE1-dependent cleavage of IR (Fig. S1). With respect to other BACE1 substrates: Neuregulin1 (NRG1) was less effectively cleaved by BACE1_R42G_ than by wildtype BACE1, whereas the amyloid precursor protein (APP) and its mutated form APPswe were similarly cleaved by both BACE1 forms (Fig. S2A). These results show that IR cleavage does not necessarily require the PC-dependent maturation of proBACE1, but they do not rule out that mature BACE1 may also cleave IR.

The cleavage of IR by proBACE1 implies that it takes place in the early secretory pathway (ESP), from endoplasmic reticulum (ER) and before the *trans* Golgi network (TGN) where the PC cleaves BACE1 prodomain, whereas mature BACE1 would cleave IR from TGN and beyond. We therefore analyzed the BACE1-dependent cleavage of IR mutated forms that are sequestered in the ESP. We have previously described the ESP sequestration of an IR mutated form that resists BACE1 cleavage, in which amino acids flanking the scissile bond are substituted (F_942_K/Y_943_K giving IR_FY/KK_) (12). Figure 2*C* confirms the ESP retention of IR_FY/KK_ (mainly IR_FY/KK_ precursor was detected) and its resistance to BACE1 cleavage (small amount of IR_CTF_ was detected). The construct IR_FY/AA_ was also retained in the ESP, but, unlike IR_FY/KK_, it was effectively cleaved by BACE1 as significant amounts of IR_CTF_ (Fig. 2*C*) and IR_SOL_ (Fig. 2*D*) were generated. These results support that BACE1 can cleave IR in the ESP where both proteins exist in their precursor forms. They also imply the existence of an N-terminal fragment of proIR (IR_NTF_) contained in intracellular vesicles, which once secreted is identified as IR_SOL_ (Fig. S1). In lysates of active BACE1-expressing cells, anti-IRα subunit antibodies detected a protein below the position of IR_FY/AA_ precursor, suggesting that this protein is the BACE1-generated IR_NTF_ (Fig. 2, *C* and *D*). SDS-PAGE migration distances of IR_FY/AA_ precursor and its IR_NTF_ were increased by EndoH treatment of the cell lysates (Fig. 2*E*), which removes glycans from core glycosylated proteins that have not trafficked to the *cis*-medial Golgi apparatus, showing that these two proteins are present within the ESP.

The IR_NTF_ generated in the ESP cisternae should be a single polypeptide chain consisting of the entire IRα subunit and a fragment of the IRβ subunit, which, once in the TGN, should be cleaved by PC (Fig. S1). Inhibition of PC (by EK4 overexpression (15) or pharmacological treatment) reduced the proteolytic maturation of IR (increased amount of proIR, decreased amount of IRβ) and BACE1 (increased detection of proBACE1) but did not impair the BACE1-dependent cleavage of IR as judged by the detection of IR_CTF_ and IR_SOL_ (Fig. 2*F*). However, IR_SOL_ generated under PC inhibition showed a reduced mobility on SDS-PAGE, which is consistent with an IR_NTF_ that is not cleaved by PC.

Of interest, fractionation of the subcellular membrane vesicles of HepG2 hepatoma cells by iodixanol density gradient ultracentrifugation allowed the detection of proIR, IR_CTF_, and IR_NTF_ in the Golgi apparatus and ER-enriched fractions (Fig. 2*G*), suggesting that cleavage of endogenous IR occurs in the ESP. However, as expected for transmembrane proteins transported through the secretory pathway, IR_CTF_ and IRβ have similar sedimentation profiles. IR_NTF,_ which is an intravesicular fragment of proIR, was narrowly detected in Golgi apparatus and ER-enriched fractions.

Taken together, these results provide arguments in favor of a cleavage of proIR by proBACE1 that occurs in ESP.

### Degradation-dependent regulation of proBACE1 amount

**Figure 3:**
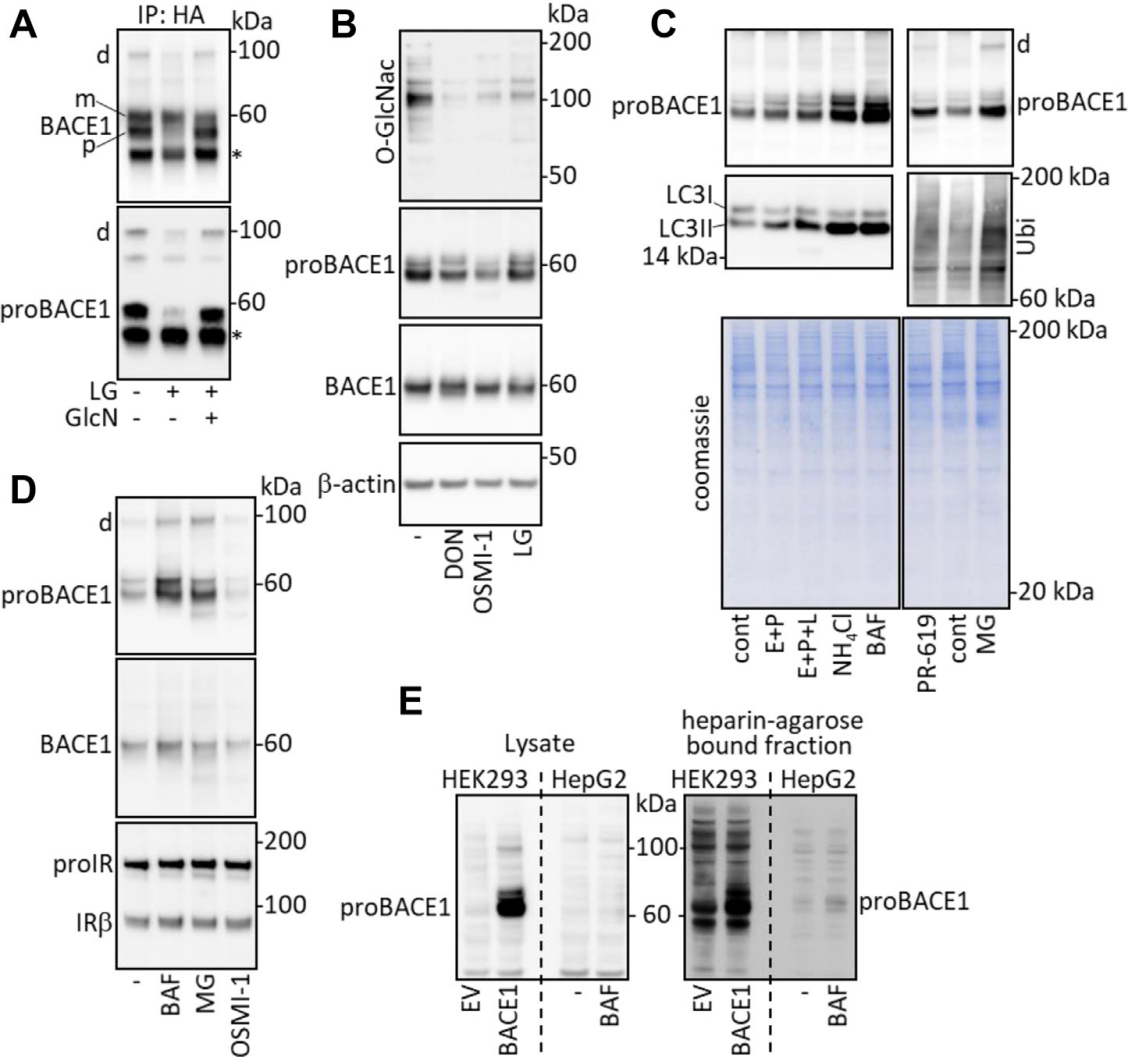
**proBACE1 proteostasis is regulated by *O*-GlcNacylation-dependent degradations.** HA-BACE1-expressing cells were (*A*) cultured in conventional media (25 mM glucose) or incubated for 17 h in low-glucose media (LG; 5.5 mM glucose) in the absence or presence of glucosamine (GlcN; 1 mM), then BACE1 was immunoprecipitated (IP: HA) and BACE1 and proBACE1 were detected, (*B*) treated for 17 h with deoxynorleucine (DON; 5 mM), OSMI-1 (35 μM), or incubated in low-glucose media (LG), then total protein *O*-GlcNacylation (O-GlcNac), proBACE1, BACE1, and β-actin (loading control) were detected in cell lysates, (*C*) left untreated (cont) or treated for 17 h with inhibitors of lysosome-dependent degradation: E-64-d (E; 10 μM) + pepstatin (P; 20 μM), E + P + leupeptin (L; 20 μM), NH_4_Cl (20 mM), Bafilomycin A1 (BAF; 30 nM) or with inhibitors of proteasome-dependent degradation: MG132 (MG; 1 μM) or PR-619 (10 μM), then proBACE1 was detected. The positive autophagy marker LC3II and total Ubiquitin were detected to control the efficacy of the inhibitors. Coomassie *blue* staining of the gels confirms that comparable amounts of proteins were analyzed. *D*, cells expressing BACE1 and IR were treated with Bafilomycin A1, MG132, or OSMI-1, then proBACE1, BACE1, and IRβ subunit were detected. *E*, HEK293 cells were transfected with empty plasmid (EV) or BACE1-expressing vector, HepG2 cells were treated for 17 h with Bafilomycin A1 (BAF; 30 nM), then proBACE1 was detected in cell lysates and in heparin-agarose bound fraction. Positions of BACE1 dimer (d), proBACE1 (p), mature BACE1 (m), heavy chain immunoglobulin (∗), and IR precursor (proIR) are indicated.

We have previously observed that proBACE1 amount and IR cleavage were regulated by the glucose concentration of the cell culture media and have suggested that the hexosamine biosynthetic pathway (HBP) plays a key role in this process (12). In agreement with this proposal, proBACE1 amount was reduced when cells were incubated in low-glucose media, an effect prevented by the addition of the HBP substrate glucosamine (Fig. 3*A*). Furthermore, low-glucose media or reduction of *O*-GlcNAcylation by inhibiting either L-glutamine-D-fructose 6-phosphate amidotransferase (the HBP rate-limiting enzyme) by DON or *O*-GlcNAc transferase (the enzyme that attaches a GlcNAc moiety on specific proteins) by OSMI-1 (16) reduced global *O*-GlcNacylation and decreased the amount of proBACE1 without major alterations of the amount of mature BACE1 (Fig. 3*B*). These results confirm that *O*-GlcNacylation controls proBACE1 proteostasis. We therefore investigated the mechanisms responsible for proBACE1 degradation.

proBACE1 amount was increased by treatment of cells with inhibitors of lysosome-dependent proteolysis, which increased the accumulation of the autophagosomes marker LC3II, and by inhibitors of proteasome-dependent proteolysis, which increased the accumulation of ubiquitinated proteins (Fig. 3*C*). Bafilomycin A1 (BAF; a V-ATPase inhibitor that increases lysosomal pH preventing lysosomal proteases activity) and MG132 (which blocks the proteolytic activity of the 26S proteasome complex) were used for the remainder of the study. Both inhibitors increased proBACE1 levels and less markedly those of mature BACE1 (Fig. 3, *C* and *D*). Comparatively, these inhibitors poorly altered the amount of precursor and mature IR (Fig. 3*D*). The proBACE1 stable dimer was also increased by BAF and MG132 treatment (Figs. 3, *C* and *D*). Treatment of HepG2 hepatoma cells with BAF increased the amount of endogenous proBACE1 (Fig. 3*E* and Fig. S4*B*), indicating that the regulation observed with overexpressed BACE1 is valid for endogenous BACE1. In HepG2 cells, endogenous proBACE1 was poorly expressed and its detection required its preliminary enrichment by heparin-agarose-based affinity chromatography, as previously described (17). These results demonstrate that the amount of proBACE1 contained in ESP is regulated by proteasome- and/or lysosome-dependent proteolysis, implying the involvement of ER-associated degradation (ERAD) and/or ER-to-lysosome-associated degradation (ERLAD) pathways (18, 19) (thereafter abbreviated as ER(L)AD).

### Ubiquitin-independent degradation of proBACE1

**Figure 4:**
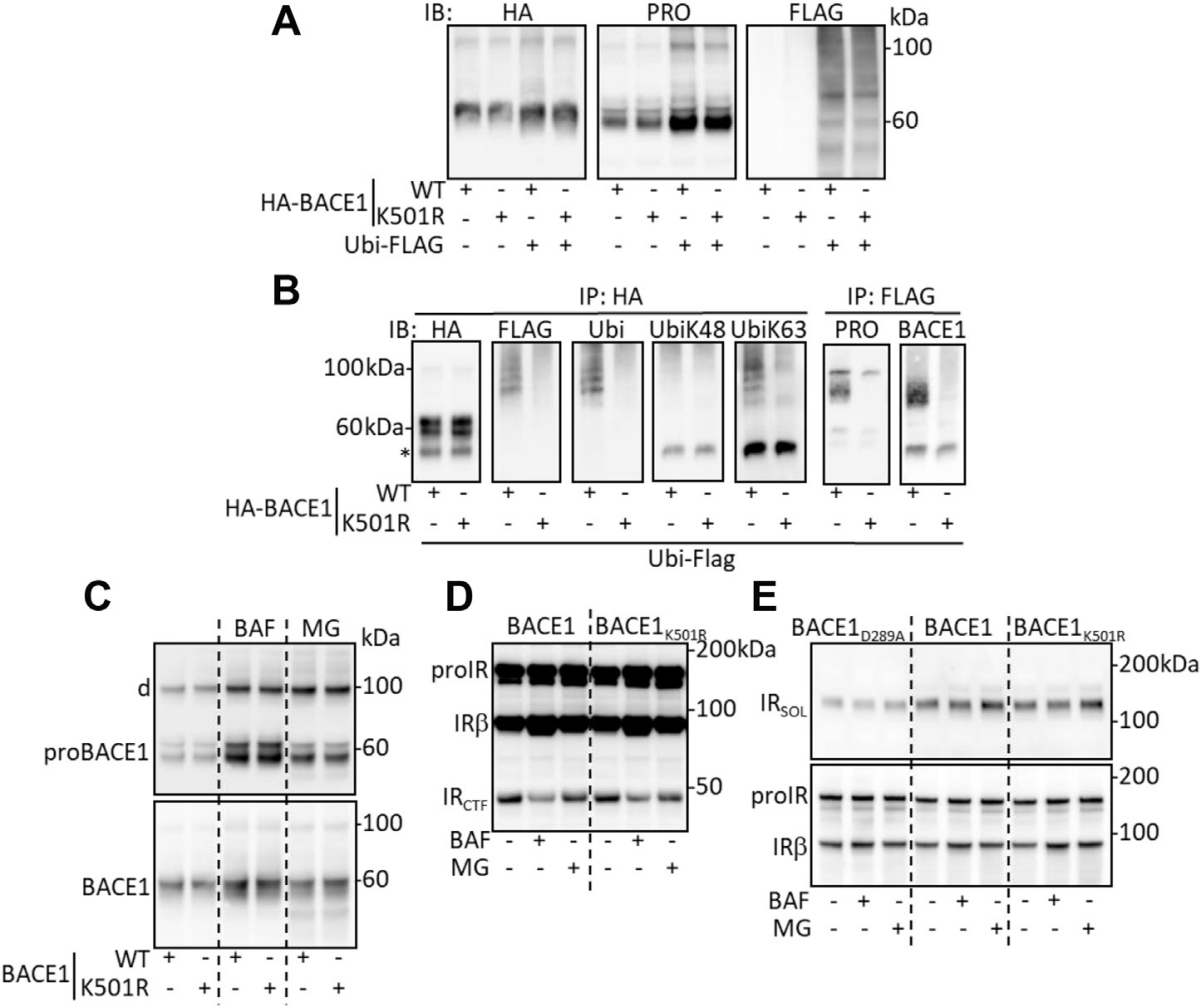
**proBACE1 is degraded by ubiquitin-independent pathway.** Cells were transfected with expression vectors coding for the wildtype (WT) or ubiquitination-deficient (K501R) BACE1 and for ubiquitin (Ubi-FLAG). *A*, detection of BACE1 (HA), proBACE1 (PRO), and ubiquitin (FLAG) in cell lysates. *B*, immunoprecipitation of BACE1 (IP: HA) or ubiquitin (IP: FLAG) followed by the detection (IB) of total ubiquitin (FLAG, Ubi), K48- and K63-linked polyubiquitin chains (UbiK48, UbiK63), proBACE1 (PRO), and BACE1. *C*, cells expressing the indicated forms of BACE1 were treated for 17 h with Bafilomycin A1 (BAF; 30 nM) or MG132 (MG; 1 μM), then proBACE1 and BACE1 were detected in cell lysate. *D*, cells expressing IR and the indicated forms of BACE1 were treated as in (*C*) in the presence of DAPT and IRβ subunit was detected in cell lysate. *E*, same as (*D*) but without DAPT treatment, IR_SOL_ and IRβ subunit were detected in the conditioned media and in cell lysates, respectively. Positions of IR precursor (proIR), IR C-terminal fragment (IR_CTF_), BACE1 dimer (d), and heavy chain immunoglobulin (∗) are indicated.

BACE1 was previously shown to be ubiquitinated at lysine 501 (20). We therefore analyzed the involvement of this posttranslational modification (PTM) in the regulation of proBACE1 degradation. Basal expression of proBACE1 and proBACE1_K501R_ (that cannot be ubiquitinated) were similar (Fig. 4*A*), and ubiquitin overexpression, which leads to the accumulation of ubiquitinated proteins that overloads the degradation systems and slows down proteolysis, greatly increased the amounts of proBACE1 and proBACE1_K501R_ and minimally those of total BACE1 (Fig. 4*A*). Immunoprecipitation of BACE1 followed by ubiquitin detection confirmed the ubiquitination of BACE1 at lysine 501 by K63-linked polyubiquitin chains (Fig. 4*B*), and immunoprecipitation of FLAG-tagged ubiquitin followed by the detection of proBACE1 revealed the ubiquitination of proBACE1 (Fig. 4*B*). Therefore, our results are in favor of a K63-linked ubiquitination of pro and mature BACE1. BACE1 lysine 501 was also shown to be a site for SUMO1 conjugation (21). However, our results are not in favor of incorporation of the nondeconjugatable SUMO1_Q89P_ into BACE1 (Fig. S3).

Basal levels of pro and total BACE1 and BACE1_K501R_ were comparable and were similarly increased by inhibition of lysosome- or proteasome-dependent proteolysis (Fig. 4*C*). Furthermore, IR was similarly cleaved by overexpressed BACE1 and BACE1_K501R_ as judged by the generation of IR_CTF_ (Fig. 4*D*) and the release of IR_SOL_ (Fig. 4*E*). The IR cleavage was distinctly affected by treatment with BAF; indeed, the IR_CTF_ was reduced (Fig. 4*D*); however, IR_SOL_ remained unchanged (Fig. 4*E*).

Therefore, PTM of BACE1 at lysine 501 is not involved in the regulation of proBACE1 levels by the two major types of protein degradation pathways. These results support the ubiquitin-independent degradation of proBACE1 by ER(L)AD systems.

### ER stress regulates proBACE1 amount

The involvement of ER(L)AD in proBACE1 proteostasis suggests that proBACE1 amount may be regulated by ER stress.

**Figure 5:**
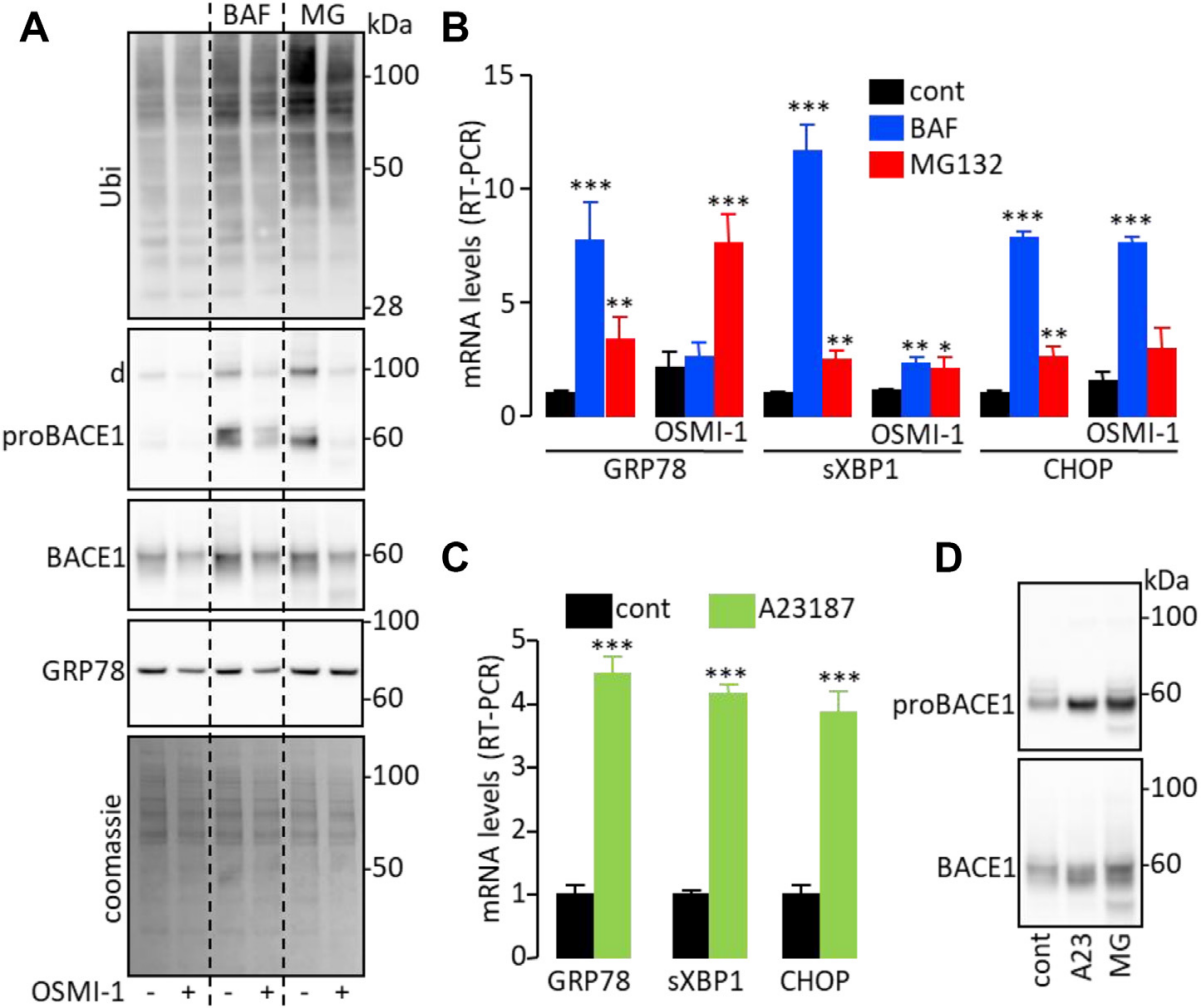
**proBACE1 degradation and ER stress are functionally connected.** Cells expressing BACE1 were treated for 17 h with Bafilomycin A1 (BAF; 30 nM) or MG132 (MG; 1 μM) along with OSMI-1 (35 μM) as indicated. *A*, total ubiquitination (Ubi), proBACE1, BACE1, GRP78, and total protein content (Coomassie staining) were detected in cell lysates (d indicates the position of BACE1 dimer). *B*, mRNA levels of ER stress markers GRP78, CHOP, and spliced XBP1 (sXBP1) were measured by RT-PCR. BACE1 overexpressing cells were treated for 17 h with A23187 (A23; 1 μM) or MG132 (MG; 1 μM), then (*C*) mRNA levels of the indicated ER stress markers were measured by RT-PCR and (*D*) proBACE1 and BACE1 were detected in cell lysates. Data are means ± SD. Statistical analyses were made using ANOVA followed by Dunnett’s (*A*) or *t* test (*C*): ∗∗∗*p* < 0.001.

In addition to increasing proBACE1 levels, inhibition of ER(L)AD systems by treatment with BAF or MG132 allowed the accumulation of ubiquitin-conjugated proteins (Fig. 5*A*) that can ultimately trigger ER stress. Coherently, BAF and MG132 increased mRNA levels of ER stress markers (Fig. 5*B*). The increase in proBACE1 levels and ubiquitin-conjugated protein accumulation triggered by BAF and MG132 were partially prevented by simultaneous treatment with OSMI-1 (Fig. 5*A*), which decreases *O*-GlcNacylation. Of interest, OSMI-1 also prevented the increase in mRNA levels of the ER stress markers GRP78 and sXBP1 induced by BAF but not by MG132 (Fig. 5*B*) and reduced the amount of GRP78 protein under basal and BAF-treated conditions (Fig. 5*A*). Remarkably, CHOP mRNA levels were not altered by OSMI-1 treatment (Fig. 5*B*).

These results suggest that ER(L)AD (probably more specifically ERLAD), *O*-GlcNacylation process, ER stress, and the regulation of proBACE1 amount are somehow functionally interrelated. In favor of this interpretation, treatment of cells with the ER stress inducer A23187 (22) upregulated the expression of ER stress markers (Fig. 5*C*) and increased the amount of proBACE1 (Fig. 5*D*).

### Regulation of proBACE1 in the liver of diabetic mouse models

**Figure 6:**
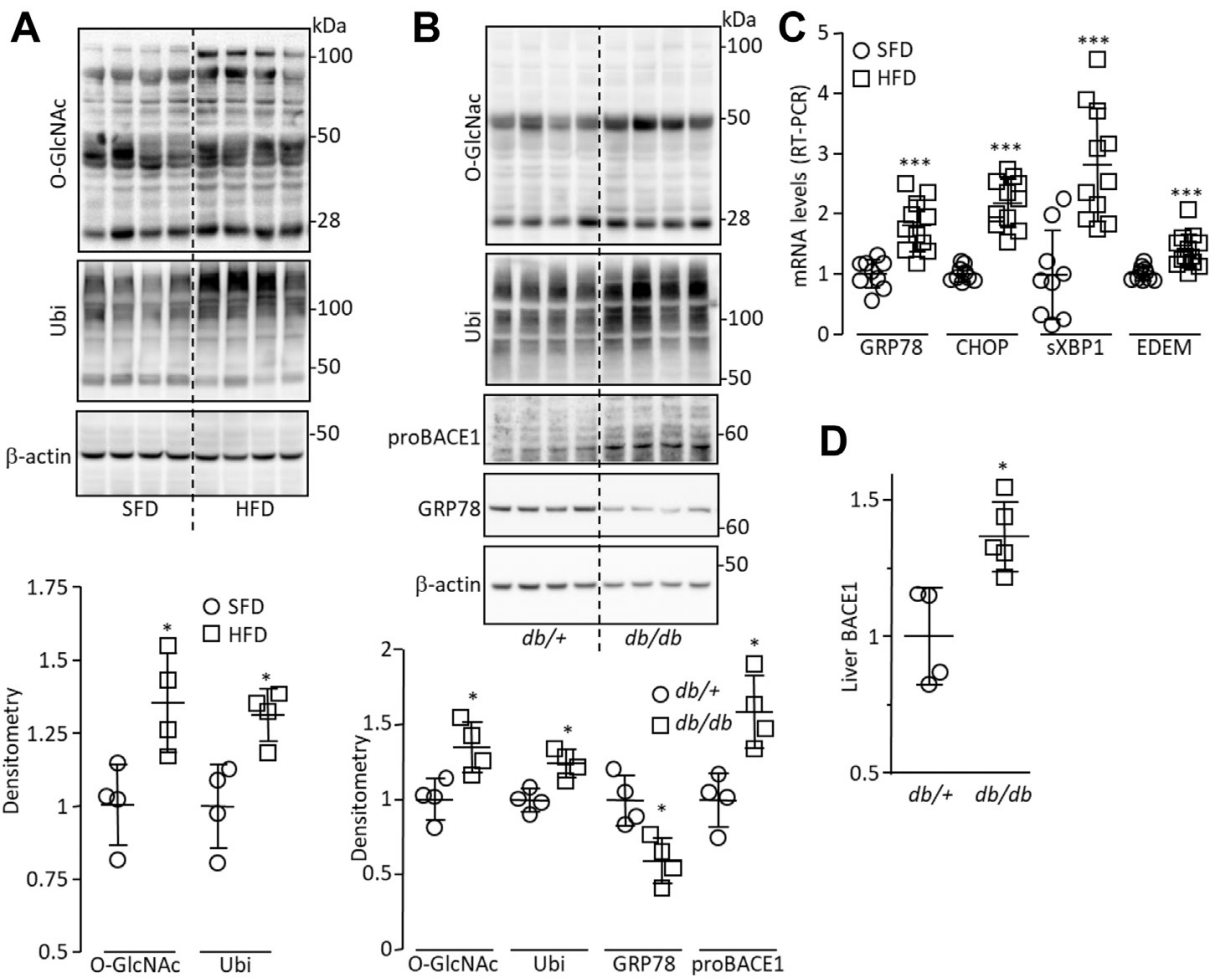
***O*-GlcNacylation, ubiquitination, and ER stress are increased in livers of *db/db* mice.***A*, *O*-GlcNacylation (O-GlcNac), total ubiquitination (Ubi), and β-actin (loading control) were detected by immunoblot in lysates of liver from standard-fat diet–fed mice (SFD) and high-fat diet–fed mice (HFD); a densitometric analysis of the signals is shown (*lower panel*). *B*, *O*-GlcNacylation, total ubiquitination, proBACE1, GRP78, and β-actin were detected in lysates of liver from *db/+* and *db/db* mice; a densitometric analysis of the signals is shown (*lower panel*). *C*, mRNA levels of the indicated ER stress markers were measured by RT-PCR, in livers from SDF- and HFD-fed mice. *D*, BACE1 was measured by ELISA in lysates of liver from *db/+* and *db/db* mice (same amount of protein was used). Values are expressed as fold over the means of the control mice. Data are means ± SD. Statistical analyses were made using Mann–Whitney (*A*, *B*, and *D*) or *t* test (*C*): ∗*p* < 0.05, ∗∗∗*p* < 0.001.

Global protein *O*-GlcNacylation and ubiquitination were increased in livers of mice fed a high-fat diet (HFD) compared with standard-fat diet–fed mice (Fig. 6*A*) as well as in those from *db/db* mice compared with their control (*db/+*) littermates (Fig. 6*B*). ER stress was increased in livers of HFD-fed mice and *db/db* mice, as indicated by the increased mRNA levels of some ER stress markers (Fig. 6*C*) and reduced amount of GRP78 protein (Fig. 6*B*), respectively. Therefore, the regulatory events that control the amount of proBACE1 (*O*-GlcNacylation, ER(L)AD, and ER stress) are disturbed in the liver of mouse models of diabetes. As expected, the amount of proBACE1 was increased in the liver of *db/db* mice (Fig. 6*B* and Fig. S4, *A* and *B*), which is in accordance with the increased BACE1 expression (12) (Fig. 6*D* and Fig. S4*C*).

### Claramine reduces BACE1-dependent cleavage of IR

**Figure 7:**
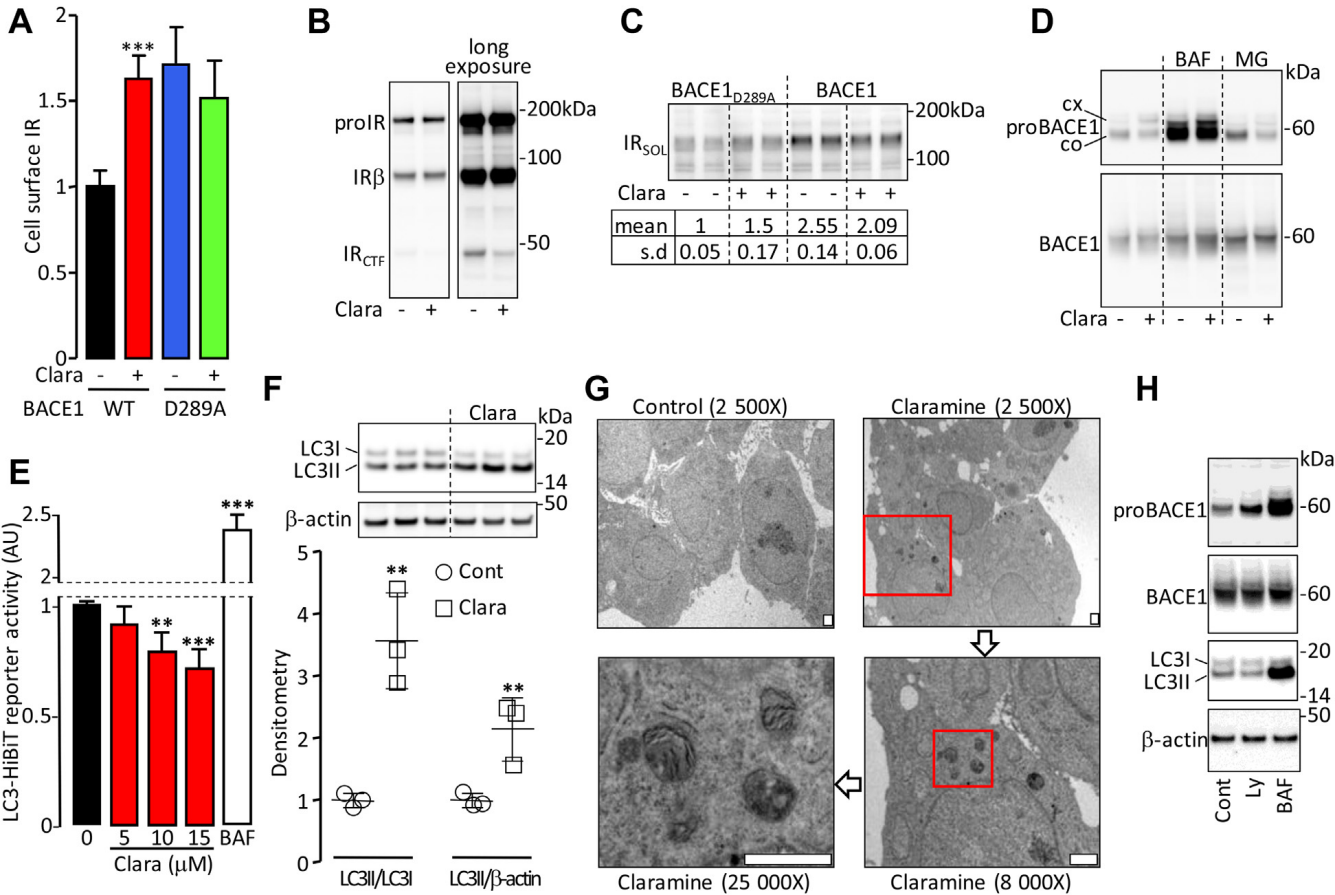
**Claramine reduces BACE1-dependent cleavage of IR.***A*, HEK293 cells expressing IR and wildtype (WT) or inactive (D289A) BACE1 were treated with Claramine (Clara; 10 μM, 17 h), then cell surface IR was measured by flow cytometry. Data are means ± SD of median fluorescence intensities corrected for the value obtained with cells transfected with the empty vector and expressed relative to the control situation (set at 1). *B*, HEK293 cells expressing BACE1 and IR were treated with Claramine (Clara) in the presence of DAPT, and IRβ subunit was detected in cell lysates. *C*, same as (*A*) except that IR_SOL_ was detected in the conditioned media; densitometric analysis of the signals was performed (*lower panel*), and values are expressed relative to the mean in the control situation. *D*, HEK293 cells expressing BACE1 were treated with Claramine (Clara), Bafilomycin A1 (BAF), or MG132 (MG), then proBACE1 and total BACE1 were detected in cell lysates. *E*, HepG2 cells transfected with LC3-HiBiT reporter were treated for 17 h with the indicated concentrations of Claramine (Clara) or with Bafilomycin A1 (BAF; 20 nM; 6 h) as positive control for autophagy inhibition. Data are means ± SD of reporter activity measured as LC3-HiBiT luminescence normalized to untreated situation. *F*, detection of LC3 and β-actin (loading control) in HepG2 cells treated with Claramine; densitometric analysis of the signals was performed (*lower panel*), and values are expressed relative to the mean in the control situation. *G*, detection by transmission electron microscopy of autophagosome-like structures (multimembranous autophagic vacuoles) in HepG2 cells treated with Claramine. The area outlined in *red* in the image is enlarged in the following image; the *white bar* at the *bottom right* of each micrograph corresponds to 1 μM. *H*, HEK293 cells expressing BACE1 were treated for 17 h with LY294002 (LY; 10 μM) or Bafilomycin A1 (BAF, 30 nM), then proBACE1, total BACE1 and LC3, and β-actin (loading control) were detected in cell lysates. Statistical analyses were made using *t* test (*A* and *F*) or ANOVA followed by Dunnett’s (*E*): ∗∗*p* < 0.01, ∗∗∗*p* < 0.001.

Previously produced aminosterol compounds (23) (Fig. S5*A*) were screened for their ability to increase the expression of IR on the surface of cells overexpressing BACE1. To that end, luminescence generated by the previously described “IR cleavage reporter system” (12) was measured on the surface of cells expressing BACE1 (Fig. S5*B*). The most important increase in cell surface–associated luminescence was obtained with Claramine treatment (Fig. S5*C*). In agreement with this result, Claramine increased the amount of IR on the surface of cells expressing BACE1 but not on the surface of cells expressing inactive BACE1_D289A_ (Fig. 7*A* and Fig. S5*D*). In addition, Claramine reduced the generation of IR_CTF_ (Fig. 7*B*) and decreased the amount of IR_SOL_ accumulated in the conditioned media of BACE1-overexpressing cells (Fig. 7*C*). The small magnitude of IR_SOL_ reduction is likely due in part to the fact that Claramine increases the BACE1-independent IR release, as observed in the conditioned media of inactive BACE1_D289A_-overexpressing cells (Fig. 7*C*). These results suggest that the inhibition of proBACE1-dependent cleavage of IR by Claramine is responsible for the increased amount of cell surface IR. Knockdown experiments previously demonstrated that endogenous IR is cleaved by endogenous BACE1 (12) in HepG2 hepatoma cells. Treatment of these cells with Claramine reduced cellular accumulation of IR_CTF_ (Fig. S5*E*) and increased cell surface expression of IR (Fig. S5*F*), implying that endogenous IR cleavage can be reduced by Claramine. Claramine decreased the amount of APP and APPswe C-terminal fragments (Fig. S2*B*), suggesting that it reduces their cleavage and therefore that BACE1 is the target of Claramine action. In contrast, Claramine decreased the total amount of NRG1 (full-length and C-terminal fragment) (Fig. S2*B*), which is not consistent with a reduction of its cleavage. Claramine increased the amount of complex N-glycosylated proBACE1 (Fig. 7*D* and Fig. S6*A*) but did not affect proIR (Fig. 7*B*), suggesting that Claramine increases the transport of proBACE1 to the Golgi apparatus without excessive impairment of overall vesicular trafficking. In addition, Claramine reduced the amount of core glycosylated proBACE1 (Fig. 7*D* and Fig. S6*A*), an effect particularly pronounced when proteasome was inhibited (Fig. 7*D*), and which suggests that Claramine activates proBACE1 lysosomal-dependent degradation. In agreement with this, Claramine increased autophagy in HepG2 cells as demonstrated by the decreased accumulation of LC3-HiBiT reporter (Fig. 7*E*), the increase in the amount of LC3II and LC3II/LC3I ratio (Fig. 7*F*) (also observed in HEK293 cells, Fig. S6), and the formation of autophagosomes (Fig. 7*G*). Of importance, inhibition of early-stage autophagy by LY294002 (24, 25) increased the amount of proBACE1 (Fig. 7*H*), which is consistent with the involvement of autophagy in proBACE1 regulation. Remarkably, the autophagy inducer Resveratrol (26) did not increase the transport of proBACE1 to the Golgi apparatus (Fig. S6), ruling out that Claramine-induced activation of proBACE1 trafficking is solely due to the increase in autophagy it triggers.

We have previously shown that the insulin-stimulated expression of immediate-early gene *EGR1* was proportional to the amount of functional IR (12). In agreement, *EGR1* promoter activity was higher in inactive BACE1_D289A_-expressing cells than in wildtype BACE1-expressing cells (Fig. S7*A*). We therefore measured the activity of *EGR1* promoter to evaluate whether the Claramine-induced increase in cell surface IR could enhance insulin response. When functional IR was expressed, Claramine increased basal and insulin-stimulated transcription of *EGR1* with similar amplitudes whether cells expressed wildtype BACE1 or inactive BACE1_D289A_, whereas no effect was observed when the kinase dead IR_Y3F_ was expressed (Fig. S7*A*). These results suggest that Claramine directly stimulates IR signaling, which was further confirmed by showing that Claramine increased IR autophosphorylation (Fig. S7*B*). Of interest, IGF1R autophosphorylation was not increased by Claramine (Fig. S7*B*).

## Discussion

**Figure 8:**
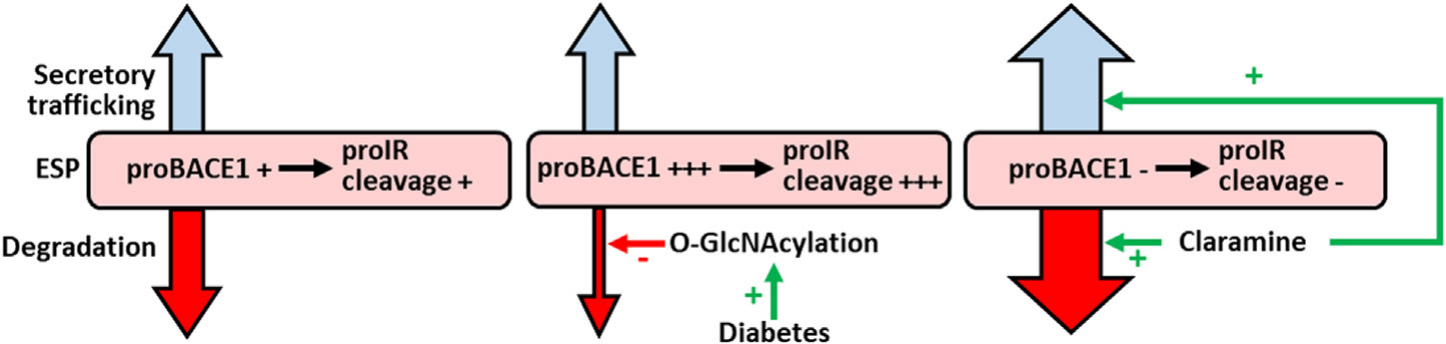
**Diagram summarizing the mechanisms that regulate IR cleavage by BACE1.** proBACE1 cleaves proIR in the early secretory pathway (ESP). The amount of proBACE1 in the ESP is regulated by its degradation (involving ER(L)AD and autophagy) and trafficking through the secretory pathway. Diabetes increases *O*-GlcNacylation, which reduces proBACE1 degradation. As a result, proBACE1 accumulates in the ESP where it cleaves proIR. Claramine increases autophagy and stimulates the trafficking of proBACE1 to the Golgi apparatus, which reduces the amount of proBACE1 in the ESP thereby decreasing proIR cleavage.

Mechanisms that regulate the cleavage of IR by BACE1 in the liver during diabetes may constitute targets to improve insulin sensitivity. Our results demonstrate that the decrease in ER(L)AD activity triggers the accumulation of proBACE1 in ESP, which leads to proIR cleavage. This cascade of reactions, which can be initiated by an increased *O*-GlcNacylation, is reversed by Claramine (Fig. 8).

We have compiled arguments demonstrating that proBACE1 can cleave proIR in the ESP. First, proBACE1 interacts with proIR. Second, lowering PC activity reduces BACE1 and IR maturation without decreasing BACE1-dependent IR cleavage; in agreement, BACE1_R42G_ that cannot be proteolytically matured by PC still cleaves IR. Third, IR_FY/AA_ that resides in the ESP as a precursor form is cleaved by BACE1 and BACE1_R42G_. Fourth, IR_NTF_ is generated by BACE1 in the ESP (as attested by its sensitivity to EndoH deglycosylation). Fifth, endogenous IR_NTF_ cosediments with the Golgi apparatus and the ER during fractionation of subcellular membrane vesicles. The ability of proBACE1 to cleave APP in the ESP has already been described (13, 27), and we confirm that the presence of the prodomain in BACE1_R42G_ does not markedly alter APP and APPswe cleavage. On the opposite, NRG1 is less cleaved by BACE1_R42G_ than by BACE1, showing that the cleavage of specific substrates can be hindered by the presence of BACE1 prodomain. We detected a dimeric form of proBACE1 that interacts with IR and whose amount seems proportional to the amount of proBACE in the ESP. Of interest, it was proposed that BACE1 dimerization would facilitate binding and cleavage of physiological substrates (28).

We have highlighted the involvement of the *O*-GlcNacylation process in the regulation of proBACE1 amount. *O*-GlcNAcylation is known to control proteasome activity (29, 30) and autophagic flux (31, 32). Our results confirm these findings since inhibition of *O*-GlcNacylation reduces global protein ubiquitination in basal situation and upon inhibition of proteasome or lysosome/autophagy. We propose that, when lysosome/autophagy is inhibited, a reduction in *O*-GlcNacylation increases proteasomal degradation; inversely, when proteasome activity is inhibited, the reduced *O*-GlcNacylation stimulates degradation, lysosomal-dependent degradation. This interpretation is consistent with the prevailing theory that considers proteasome and autophagy as two complementary and mutually regulated protein degradation systems (33). Inhibition of proteasome- or lysosome/autophagy-dependent degradations induces a substantial increase in proBACE1 amount, which is prevented by inhibition of *O*-GlcNacylation. The involvement of proteasome (34, 35, 36) or lysosome (20, 37) in the regulation of BACE1 degradation has already been reported. Increased amounts of proBACE1 were observed when Ubiquitin carboxyl-terminal hydrolase L1 is knocked down (38) or when lysosome is inhibited (37). Therefore, our results showing that proteasome or lysosome inhibition increases the amount of proBACE1 are consistent with these earlier unexplained observations. In addition, we show that an inhibitor of early-stage autophagy increases proBACE1 amount, suggesting a direct role of autophagy in this regulation.

The K48-linked polyubiquitin chain has been shown to be sufficient to target substrates to the 26S proteasome, whereas the K63-linked polyubiquitin chain is involved in various stages of internalization, lysosome sorting, and degradation processes of membrane proteins (39, 40). However, this concept is oversimplified and exceptions occur. For instance, both K63 and K48 ubiquitin linkages signal lysosomal degradation of the LDL receptor (41). We confirm that BACE1 is ubiquitinated at lysine 501 by K63-linked polyubiquitin chain (20) and show that proBACE1 is also subject to this PTM. There is no consensus regarding the stability of the ubiquitination-deficient BACE1_K501R_: it was shown to behave like wildtype BACE1 (42) or to be stabilized and accumulate in early and late endosomes/lysosomes (20) or to be destabilized (21). In our experimental setting, proBACE1 and BACE1 amounts are not altered by the substitution of the ubiquitination site, either in the basal situation or during proteasome or lysosome inhibition, suggesting that BACE1 ubiquitination is not involved in the control of its degradation and therefore that BACE1 and proBACE1 undergo ubiquitin-independent degradation. Such a degradation process was already described for other proteins (43, 44). The localization of proBACE1 in the ESP implies that its degradation is regulated by processes controlling protein degradation in ER, *i.e.*, ER(L)AD pathways (18, 19), which are known to be triggered by ER stress. Inhibition of ER(L)AD pathways increases ER stress, likely by creating a protein overload in ER. Of interest, the ER stress inducer A23187 increases the amount of proBACE1, suggesting that, in our study, the unfolded protein response is not sufficient to restore adequate proteostasis of proBACE1. This result shows that ER stress may be a trigger for disruption of proBACE1 proteostasis. We confirm, as previously described (45, 46, 47), that *O*-GlcNacylation, ubiquitination, and ER stress are increased in the liver of mouse models of diabetes and we further show that these abnormalities accompany an increase in the amount of proBACE1 and total BACE1. Our interpretation is that, during diabetes, hyperglycemia enhances *O*-GlcNacylation, which reduces ER(L)AD (both contributing to ER stress), allowing the abnormal accumulation of proBACE1 in the ESP, which promotes the proBACE1-dependent cleavage of IR precursor.

The aminosterol compound Claramine and its analogue Trodusquemine are PTP1B inhibitors (48, 49) that prevent IR dephosphorylation, keeping it in its activated state. Our results confirm that Claramine increases IR phosphorylation and signaling. Of interest, IGF1R phosphorylation, which is also controlled by PTP1B (50), is not increased by Claramine, which suggests that Claramine has very special properties resulting in a “specific” increase in IR phosphorylation. Of the aminosterols tested, only Claramine increases IR cell surface expression, and this implies its ability to inhibit IR cleavage by BACE1. Claramine also reduces the BACE1-dependent cleavages of APP and APPswe (that are efficiently cleaved by proBACE1) but does not seem to impact NRG1 cleavage (that is poorly cleaved by proBACE1), suggesting that Claramine alters proBACE1 biology. Claramine reduces the amount of proBACE1 in the ESP. As this effect is particularly pronounced when proteasome is inhibited, we propose that it is due to Claramine’s ability to increase the autophagy. However, this increased autophagy alone cannot fully explain the selective reduction of proBACE1 amount. Indeed, Claramine does not affect all the proteins overrepresented in the ESP, as shown by its lack of effect on proIR. Moreover, the autophagy inducer Resveratrol (26) does not reduce the amount of proBACE1. Claramine increases the amount of proBACE1 in the Golgi apparatus, suggesting that it increases the efficiency of proBACE1 trafficking from the ESP to the Golgi apparatus. Genetic alterations of autophagic process in mice revealed that autophagy reduction in the liver participates to insulin resistance development (51, 52, 53). Our view (see above) is that reduced autophagy is one of the triggers for the cleavage of proIR by proBACE1, which worsens insulin resistance (12). In this context, Claramine through its abilities to increase autophagy, to reduce the cleavage of IR by BACE1, and to inhibit PTP1B could be a high-potential compound to improve the liver insulin sensitivity. As a matter of fact, Claramine has already shown its efficacy to improve glycemic control in diabetic mice (49, 54). The properties of Claramine also make it an attractive molecule in the fight against Alzheimer disease (AD). Indeed, BACE1 (55) and defects in autophagy (56) are involved in AD pathogenesis, and PTP1B inhibitors are considered a promising strategy to combat a variety of AD-related detrimental processes (57). It then appears necessary to better delineate the biological effects of Claramine and to elucidate its mechanisms of action.

In summary, we characterized the sequence of events leading to the cleavage of IR by BACE1 in the liver during diabetes. We identified Claramine as an efficient antagonist of proBACE1-dependent cleavage of proIR with modes of action compatible with its use as a hepatic insulin sensitizer.

## Experimental procedures

### Chemicals

DAPT and dec-RVKR-cmk were from Merck. DON, OSMI-1, PUGNAc, glucosamine, insulin, heparin agarose, A23187, LY294002, and IGF2 were from Sigma-Aldrich. Bafilomycin A1, MG-132, PR-619, Leupeptin, E-64-d, and pepstatin-A were from Santa Cruz Biotechnology

### Antibodies

Antibodies for IR (C-19 for β-subunit and H-78 for α-subunit), anti-phosphorylated IR (Tyr^1162/1163^) that also detects phosphorylated IGF1R (Tyr^1135/1136^), BACE1 (61-3E7), GRP78 (76-E6), *O*-GlcNac (CTD110.6), HA-probe (Y-11), and c-Myc (9E10) antibodies were from Santa Cruz Biotechnology. Golgin 97 monoclonal antibody (CDF4) was from (ThermoFisher Scientific). Antibodies for β-actin (13E5), GAPDH (D4C6R), Ubiquitin (P4D1), K63- and 48-linkage specific polyubiquitin (D7A11 and D9D5), and LC3 were from Cell Signaling Technology. BACE1 prodomain antibody (26, 27, 28, 29, 30, 31, 32, 33, 34, 35, 36, 37, 38, 39, 40, 41, 42, 43, 44, 45) was from Genscript. Anti-FLAGM2 was from Sigma-Aldrich. IGF1R antibody (NBP2-24885) was from Novus Biologicals. Human IR Alexa Fluor 488–conjugated Antibody (clone 243522), used in flow cytometry experiments, and Human BACE1 ELISA kit were from R&D Systems.

### Expression vectors

Expression vectors for human NRG1 and APP were from OriGene Technologies. Expression vector for HA-SUMO1_Q94P_ was a gift from G. Salvesen (Addgene plasmid # 48965), Ubiquitin-FLAG and IGF1R expression vectors were from K. L. Lim (Lee Kong Chian School of Medicine) and R. O'Connor (University College Cork), respectively. *EGR1* promoter-luciferase reporter vector was from J. L. Jameson (Northwestern University). Expression vectors for IR, IR_FY/KK_, IR_Y3F,_ IRβ N-terminally fused to Gaussia luciferase, furin, EK-4, HA-tagged BACE1 inactive BACE1_D289A_ were previously described (12). Mutated forms of BACE1 and IR and APPswe (the Swedish mutation K595N/M596L) were created using the GeneArt site-directed mutagenesis system (Thermo Fisher Scientific) or In-fusion cloning system (Takara Bio).

### Cell culture and transfection

HEK293 cells (Griptite 293 MSR) were from Thermo Fisher Scientific. HepG2 cell lines were from American Type Culture Collection. Cells were maintained in culture as described by the manufacturers. No mycoplasma contamination was detected in any of the cultures. Transfections were performed with PolyJet reagent (SignaGen Laboratories), as specified by the manufacturers.

### Mice

Male *db/db* (*BKS.Cg-m+/+Leprdb/J*) and *db/+* (*BKS.Cg-m+/−Leprdb/J*) mice (Charles River Laboratories) were 10 weeks old at the time of killing. Liver was removed, and pieces were snap frozen for ulterior analysis. Ten-week-old male C57Bl/6 mice (Charles River Laboratories) were randomly fed either a standard-fat diet (70% kcal carbohydrate, 10% kcal fat, 20% kcal protein, 3.68 kcal g^−1^, Special Diet Services) or a HFD (20% kcal carbohydrate, 60% kcal fat, 20% kcal protein, 5.13 kcal g^−1^) for 16 weeks before killing. Blood and tissues were collected as described above. Experiments were performed at Aix Marseille University, France, in accordance with the European directive 2010/63/EU on the protection of animals used for scientific purposes and approved by the “Comité d'éthique en expérimentation animale de Marseille.”

### Enzymatic deglycosylation

N-linked carbohydrate residues were removed by incubating cell lysates for 2 h at 37 °C with 1000 units of endoglycosidase H (EndoH) as described by the manufacturer (New England Biolabs). Samples were then separated by SDS-PAGE and analyzed by Western blotting.

### Subcellular fractionation

The fractionation was performed using iodixanol gradient (Sigma-Aldrich). HepG2 cells were cultured for 36 h in the presence of 25 mM glucose and treated with 10 μM DAPT for the last 17 h then washed and scraped in homogenization buffer (0.25 M sucrose, 10 mM Hepes, pH 7.4, 25 mM KCl, 2 mM MgCl_2_, 1 mM EDTA, protease inhibitor cocktail) and homogenized by 15 strokes through a 26-gauge needle syringe. The homogenate was centrifuged at 1000*g* for 10 min to obtain postnuclear supernatant. The postnuclear supernatant was further centrifuged at 10,000*g* for 10 min to obtain postmitochondrial supernatant. The postmitochondrial supernatant was further centrifuged at 100,000*g* for 2 h, and the resulting membrane pellet was suspended in 1.6 ml of homogenization buffer containing 25% (w/v) iodixanol. The vesicle suspension was layered underneath a gradient consisting of 1.6 ml of 20%, 15%, 10%, and 5% iodixanol solutions. The gradient was centrifuged using a SW41Ti rotor at 50,000*g* for 18 h, then 500 μl fractions were collected.

### Immunoblot

Cells were lysed in the presence of a cocktail of protease and phosphatase inhibitor. PUGNAc (100 μM) or PR-619 (100 μM) was added in the lysis buffer for the study of *O*-GlcNacylation or Ubiquitination, respectively. Identical amounts of total protein were heat denatured and reduced (70 °C; 10 min) then submitted to SDS-PAGE separation on 4% to 12% gradient or 12% NuPAGE gels (Thermo Fisher Scientific) and transferred to polyvinylidene fluoride membranes. Membranes were blocked for 1 h in 5% BSA solution and incubated with the appropriate primary and horseradish peroxidase–conjugated secondary antibodies (1:1000 and 1:10,000 dilutions, respectively). Immunodetections were performed using ECL reagent, and image acquisition was performed by using a chemiluminescent CCD imager ImageQuant LAS 4000 (GE Healthcare). Densitometric analysis of the bands was performed with the ImageQuant TL software.

### Flow cytometry

Cell surface expression of overexpressed IR was analyzed by flow cytometry (BD Accuri C6; BD Biosciences). Cells were gated on forward and side scatter to exclude dead cells, debris, and aggregates.

### Real-time PCR analysis

Total RNA was extracted using Nucleospin RNA Kit (Macherey-Nagel); cDNA was synthesized from 0.5 μg of RNA using M-MLV reverse transcriptase (Thermo Fisher Scientific) and used for PCR amplification. RT-PCR was performed on the LightCycler 480 instrument (Roche Applied Science) using the Eva Green MasterMix (Euromedex). The comparative Ct method (2^−(ΔΔCT)^) was used to calculate the relative differences in mRNA expression. The acidic ribosomal phosphoprotein P0 was used as housekeeping gene. Primer sequences are available upon request. Changes were normalized to the mean of control values, which were set to 1.

### Reporter assays

For gene reporter assays, HEK293 cells were transfected with *SV40*-driven Renilla luciferase vector along with *EGR1* promoter-driven Firefly luciferase in addition to BACE1 and IR expression vectors. Firefly and Renilla luciferase were measured in cell lysates after addition of proper Genofax reagents (Yelen) using a luminometer (EnSight Multimode plate reader; PerkinElmer). Activities were calculated as the ratio Firefly/Renilla luciferase and expressed as fold change compared with control. IR-cleavage reporter assay was previously described (12). For luminescence-based Autophagy reporter assay, HepG2 cells were transiently transfected with the autophagy LC3-HiBiT Reporter vector (Promega, GA2550) encoding a fusion protein consisting of human LC3B, an N-terminal 11-amino-acid HiBiT tag, and a connecting spacer that enhances reporter specificity for the autophagic pathway. Compounds to be tested were added at least 36 h after transfection for a duration of 6 h (Bafilomycin A1) or 17 h (Claramine), then LC3-HiBiT reporter activity was measured using the Nano-Glo HiBiT Lytic Detection System (Promega, N3040). A decrease in LC3-HiBiT reporter activity reflects autophagic LC3 degradation and serves as a measure of autophagy induction while an increased LC3-HiBiT reporter activity indicates autophagy inhibition.

### Transmission electron microscopy

After 5 min of wash with 0.1 M sodium cacodylate buffer, cells were directly fixed in solution with glutaraldehyde 2.5% in 0.1 M sodium cacodylate buffer for 1 h at room temperature, then washed three times for 5 min with 0.1 M cacodylate buffer. Specimens were postfixed with 2% osmium tetroxide in 0.1 M sodium cacodylate buffer for 1 h, then washed again three times for 10 min with 0.1 M sodium cacodylate buffer. Progressive dehydration was carried out with 50% to 100% ethanol bath before starting embedding in Low-Viscosity-Embedding Epoxy resin (SPURR) kit from 33% to 100% SPURR. Cells were transferred from wells to tube before polymerization of resin overnight at 72 °C. Ultrathin 60-nm sections were obtained using Ultracut-E ultramicrotome (Reichert595 Jung), and contrast was performed using Uranyl acetate and lead citrate solution. Pictures were obtained using JEM 1400 transmission electron microscope (JEOL) at 80 kV with Megaview III Camera under iTEM Five software (SIS Imaging).

### Statistical analyses

All the experiments have been repeated at least three times. Data were analyzed with GraphPad Prism software, and individual statistical two-sided tests used are identified in the figure legends. *p*-Values ≤0.05 were considered statistically significant.

## Data availability

Sequences of primers are available upon request (corresponding author). All remaining data are contained within the article.

## Supporting information

This article contains supporting information (12, 21, 58).

## Conflict of interest

The authors declare that they have no conflicts of interest with the contents of this article.
