# Supplementary material for: The aminosterol Claramine inhibits β-secretase 1–mediated insulin receptor cleavage
Source: J Biol Chem. 2021 May 23;297(1):100818. doi: 10.1016/j.jbc.2021.100818 (PMC8254121; doi:10.1016/j.jbc.2021.100818)
Supplement: Supplemental Figures S1–S7 [file mmc1.docx]

**Supporting information**

**The aminosterol Claramine inhibits β-secretase 1-mediated insulin receptor cleavage**

Bénédicte Gaborit^1,2^, Roland Govers^1^, Alexandre Altié^1^, Jean Michel Brunel^3^, Pierre Morange^1,4^, Franck Peiretti^1*^

**Material included**

Supporting Figures S1 to S7

Supporting Experimental procedures

**Figure S1: Schematic illustration of IR proteolysis during its intracellular trafficking**

In the early secretory pathway (ESP), IR precursor (proIR; a single polypeptide chain consisting of the sequences of IRα (green) and IRβ (red) subunits) is cleaved by proBACE1, generating the N- and C-terminal fragments of IR (IR_NTF_ and IR_CTF_, respectively). In the trans Golgi network (TGN), Furin cleaves proIR, generating IRα and IRβ subunits that reorganize to form mature IR. Furin cleaves also IR_NTF_ generating the soluble form of IR (IR_SOL_) that is then released in the extracellular media. γ-secretase is active in the TGN (Baulac, S., LaVoie, M. J., Kimberly, W. T., Strahle, J., Wolfe, M. S., Selkoe, D. J., and Xia, W. (2003) Functional gamma-secretase complex assembly in Golgi/trans-Golgi network: interactions among presenilin, nicastrin, Aph1, Pen-2, and gamma-secretase substrates. *Neurobiol. Dis.* **14**, 194–204) where it cleaves IR_CTF_ and generates its intracellular domain (IR_ICD_) that is degraded by the proteasome. Inhibitions of Furin by Dec-RVKR-CMK (RV) and EK4 (EK) and γ-secretase by DAPT are illustrated.

**Figure S2: Cleavage of NRG1, APP and APPswe by proBACE1, and effect of Claramine**

(**a**) HEK293 cells expressing wild-type (WT), inactive (D289A) or PC-resistant (R42G) forms of BACE1 along with C-terminal-FLAG-tagged NRG1, APP or APPswe were incubated with DAPT then full-length (FL) and C-terminal fragment (CTF) of NRG1, APP and APPswe were detected using anti FLAG antibody (lower panel: long exposure of the lower part of the blot). The BACE1-dependent cleavage of these proteins is validated by the reduced generation of their CTF in BACE1_D289A_ overexpressing cells. NRG1 was less efficiently cleaved by BACE1_R42G_ and BACE1_4CA_ than by wild-type BACE1. APP and APPswe were less efficiently cleaved by BACE1_4CA_ than by wild-type BACE1.

(**b**) Cells transfected with BACE1 along with APP, APPswe or NRG1 expression vectors were treated with Claramine then these proteins (FL) and their C-terminal fragment (CTF) generated by the BACE1-dependent cleavage were detected. Claramine reduces the amounts of the C-terminal fragments of APP, APPswe and NRG1, suggesting that it reduces their BACE1-dependent cleavage.

#

# **Figure S3: BACE1 is not SUMOylated**

# HEK293 cells were transfected with expression vectors for MYC-BACE1, the SUMO-conjugating enzyme HA-Ubc9 and HA-SUMO1_Q89P_ (that cannot be deconjugated), then HA-tagged proteins (Ubc9 and SUMO1_Q89P_) were detected in lysates and after BACE1 immunoprecipitation (IP: MYC). Positions of Ubc9 and SUMOylated RanGap are indicated.

# RanGap is a stable SUMO1-conjugated protein easily detected in HEK293 cell lysates.

# SUMOylated BACE1 was reported to have an apparent MW of 72 kDa on SDS***-***PAGE (Bao, J., Qin, M., Mahaman, Y. A. R., Zhang, B., Huang, F., Zeng, K., Xia, Y., Ke, D., Wang, Q., Liu, R., Wang, J.-Z., Ye, K., and Wang, X. (2018) BACE1 SUMOylation increases its stability and escalates the protease activity in Alzheimer’s disease. *Proc. Natl. Acad. Sci. U.S.A.* 115, 3954–3959), which is very distant from the band detected here at around 100 kDa after BACE1 immunoprecipitation.

**Figure S4: Validation of murine BACE1 detection**

(**a**) HEK293 cells were transfected with an empty vector (EV) or with vectors coding for human or mouse BACE1 (hBACE1 and mBACE1, respectively) then BACE1 and proBACE1 were detected in cell lysates, confirming that the proBACE1 specific antibody detects mouse proBACE1.

(**b**) The proper detection of proBACE1 in livers form *db/+* and *db/db* mouse was verified by showing that, after enrichment by heparin agarose chromatography, the proBACE1 signal aligned with the one increased by the treatment of HepG2 cells with Bafilomycin A1 (BAF; 35 nM; 17 hours). Coomassie blue staining of the gel confirms that comparable amounts of proteins were analyzed.

(**c**) Linearity of the detection of mouse BACE1 (mBACE1) by the commercial human BACE1 ELISA assay was confirmed by testing serial dilutions (1:10 to 1:160) of lysate of cells overexpressing mBACE1 or transfected with an empty vector (EV).

**Figure S5: Claramine increases IR cell surface expression**

(**a**) Chemical structures of some polyaminosterol compounds used in the study.

(**b**) Representation of the IR-cleavage reporter system (Meakin, P. J., Mezzapesa, A., Benabou, E., Haas, M. E., Bonardo, B., Grino, M., Brunel, J.-M., Desbois-Mouthon, C., Biddinger, S. B., Govers, R., Ashford, M. L. J., and Peiretti, F. (2018) The beta secretase BACE1 regulates the expression of insulin receptor in the liver. *Nat Commun*. **9**, 1306) (IRLuc; consisting of the Gaussia luciferase (Luc) fused to the N-terminus of IRβ-subunit) and its behavior when expressed at the surface of cells treated or not with BACE1 inhibitor. BACE1 inhibition reduces IRLuc cleavage, consequently cell surface IRLuc increases.

(**c**) Example of results obtained by screening polyaminosterol compounds (shown in A) for their ability to alter the expression of Luc on the surface of cells overexpressing IRLuc and BACE1. Cells cultured in white 96 well plates were incubated for 17 hours with 10 μM of the indicated compounds (Trodusquemine: Trod; Claramine: **4f**; Claramine A1: Clara A1) then Gaussia luciferase substrate was added to the washed cells and luminescence was recorded with a luminometer (bar graph); inset shows image of the wells acquired by CCD camera.

(**d**) Typical flow cytometry histograms corresponding to the detection of IR on the surface of HEK293 cells expressing wild-type BACE1 or inactive BACE1_D289A_ and treated with Claramine (Clara; 10 μM, 17 hours). Typical flow cytometry histograms are shown.

(**e**) HepG2 cells were treated with Claramine (Clara) and DAPT as indicated and IRβ subunit was detected in cell lysates.

(**f**) HepG2 were treated with Claramine then cell surface IR was measured by flow cytometry. Data are means ± s.d of median fluorescence intensities expressed relative to the control situation (set at 1). Statistical analyses were made using *t-test* **p < 0.01.

**Figure S6: Claramine increases proBACE1 trafficking and autophagy**

HEK293 cells expressing BACE1 were incubated for 17 hours with Claramine (Clara; 10 μM) or with the autophagy inducer Resveratrol (Res; 50 μM), then proBACE1 and the autophagy marker LC3I and LC3II and β-actin (loading control) were detected. Claramine decreases the amount of core glycosylated (co) proBACE1 and increases that of complex N-glycosylated (cx) proBACE1, suggesting an increased trafficking from ER to Golgi. Resveratrol does not increase proBACE1 intracellular trafficking. The increase in LC3II indicate that Claramine and Resveratrol increase autophagy.

**Figure S7: Claramine increases IR signaling**

(**a**) Cells were transfected with SV40-driven Renilla luciferase coding vector and the indicated combinations of expression vectors for IR, kinase dead IR_3YF_, wild-type (WT) or inactive (D289A) BACE1 along with *EGR1* promoter-driven Firefly luciferase reporter constructs. Then, cells were treated with Claramine (Clara) and/or insulin (1 nM, 7 hours) and promoter activity was measured in cell lysates and expressed as the ratio Firefly / Renilla luciferase. Stimulation of *EGR1* promoter activity by Claramine (significant p < 0.001, *t-test*) requires the presence of kinase active IR, occurs in the absence of insulin and is independent of BACE1 activity.

(**b**) Cells transfected with empty vector (EV), IR, kinase dead IR_3YF_ or IGF1R expression vector were treated with Claramine (Clara) or stimulated with insulin (50 nM; 15 min) or IGF2 (10 nM; 15 min) then IR, IGF1R, phospho IR (pIR) and phospho IGF1R (pIGF1R) were detected by immunoblot. Claramine increases the phosphorylation of IR but not that of IGF1R.

**Supporting Experimental procedures**

**Chemistry -** All solvents were purified according to reported procedures, and reagents were used as commercially available. Methanol, ethyl acetate, dichloromethane, ammonia and petroleum ether (35-60 °C) were purchased from SDS and used without further purification. Column chromatography was performed on SDS silica gel (70-230 mesh). ^1^H NMR and ^13^C NMR spectra were recorded in CDCl_3_ on a Bruker AC 250 spectrometer working at 250 MHz and 63 MHz, respectively (the usual abbreviations are used: s: singlet, d: doublet, t: triplet, q: quadruplet, m: multiplet). Tetramethylsilane was used as internal standard. All chemical shifts are given in ppm. Trodusquemine was prepared according previously reported procedure in literature.

**Synthesis of derivatives 4a-4i**

**General procedure for the titanium–mediated reductive amination reaction of 4f:** A mixture of 6-ketocholestanol (157 mg, 0.39 mmol), titanium(IV) isopropoxide (573 µL, 2.02 mmol) and spermine (202 mg, 1 mmol) in absolute methanol (5 mL) was stirred under argon at room temperature for 12 h. Sodium borohydride (38 mg, 1 mmol) was then added at -78 ºC and the resulting mixture was stirred for an additional 2 hours. The reaction was then quenched by adding water (1 mL) and stirring was maintained at room temperature for 20 minutes. The resulting inorganic precipitate was filtered off over a pad of Celite and washed with methanol and ethylacetate. The combined organic extracts were dried over Na_2_SO_4_, filtered and concentrated in vacuo to afford the expected crude amino derivative which was purified by flash chromatography affording the expected amino derivative. Purification by column chromatography (silica gel; CH_2_Cl_2_/ MeOH/ NH_4_OH(32%), 7:3:1) afforded a pale yellow solid in 45% yield; this compound can be converted subsequently into its hydrochloride salt as white solid –^1^H NMR (300 MHz, CD_3_OD) : *δ* = 0.67-0.76 (m, 4H), 0.90-1.17 (m, 24H), 1.20-1.46 (m, 7H), 1.47-1.92 (m, 20H), 2.03-2.07 (m, 1H), 2.50-2.54 (m, 1H), 2.63-2.73 (m, 13H), 3.56-3.63 (m, 1H) –^13^C NMR (75 MHz, CD_3_OD) : *δ* = 71.50, 58.96, 56.27, 56.05, 54.78, 49.98, 49.21, 47.99, 47.84, 47.34, 42.62, 40.47, 39.94, 39.49, 39.06, 36.44, 36.14, 35.86, 35.77, 35.63, 33.58, 31.61, 30.45, 28.18, 27.97, 24.37, 23.78, 22.78, 22.52, 21.03, 18.63, 16.30.12.13. C_37_H_72_N_4_O ; MS (ESI) m/z = 589.5 [M+H]^+^

**6β-(1,2-diaminoéthane)-cholestan-3β-ol** **4a:** Purification by column chromatography (silica gel; CH_2_Cl_2_/ MeOH/ NH_4_OH(32%), 7:3:1) afforded a pale yellow solid in 96% yield; – ^1^H NMR (300 MHz, MeOD): δ = 3.29-3.63 (m, 1H), 0.57-2.83 (m, 53H) – ^13^C NMR (75 MHz, MeOD): δ = 71.57, 58.79, 58.61, 56.28, 56.00, 54.74, 50.95, 47.27, 42.62, 41.88, 39.93, 39.48, 39.00, 36.23, 36.14, 36.05, 35.75, 35.64, 31.54, 30.39, 27.96, 24.36, 23.79, 22.76, 22.52, 21.03, 18.63, 15.21, 12.12. C_29_H_54_N_2_O ; MS (ESI) m/z = 447.3 [M+H]^+^

**6β-(1,4-diaminobutane)-cholestan-3β-ol 4b:** Purification by column chromatography (silica gel; CH_2_Cl_2_/ MeOH/ NH_4_OH(32%), 7:3:1) afforded a pale yellow solid in 73% yield; – ^1^H NMR (300 MHz, MeOD): δ = 0.66-3.57 (m, 58H) – ^13^C NMR (75 MHz, MeOD): δ = 71.65, 59.88, 58.54, 56.29, 56.04, 54.75, 48.18, 47.29, 42.71, 42.64, 39.94, 39.50, 39.04, 36.16, 35.78, 35.65, 31.56, 31.03, 30.40, 29.67, 27.99, 25.96, 24.35, 23.81, 22.79, 22.54, 21.05, 18.65, 16.33, 14.09, 12.15. C_31_H_58_N_2_O ; MS (ESI) m/z = 475.4 [M+H]^+^

**6β-(1-(3aminopropyl)imidazol)-cholestan-3β-ol 4c:** Purification by column chromatography (silica gel; CH_2_Cl_2_/ MeOH/ NH_4_OH(32%), 7:3:1) afforded a pale yellow solid in 64% yield; – ^1^H NMR (300 MHz, MeOD): δ = 6.87-7.44 (m, 4H), 3.96-4.03 (m, 2H), 3.56-3.63 (m, 1H), 0.56-2.70 (m, 50H) – ^13^C NMR (75 MHz, MeOD): δ = 137.14, 128.95, 118.92, 71.35, 58.93, 56.21, 55.90, 54.67, 47.14, 44.86, 44.50, 42.56, 39.84, 39.41, 38.90, 38.52, 36.07, 35.82, 35.68, 35.61, 31.67, 31.44, 30.40, 28.10, 27.91, 24.27, 23.72, 22.72, 22.47, 20.97, 18.59, 16.27, 12.06. C_33_H_57_N_3_O ; MS (ESI) m/z = 512.7 [M+H]^+^

**6β-(1-(3aminopropyl)pyrrolidine)-cholestan-3β-ol 4d:** Purification by column chromatography (silica gel; CH_2_Cl_2_/ MeOH/ NH_4_OH(32%), 7:3:1) afforded a pale yellow solid in 80% yield; – ^1^H NMR (300 MHz, MeOD): δ = 0.62-3.97 (m, 62H) – ^13^C NMR (75 MHz, MeOD): δ = 71.41, 58.74, 56.23, 56.01, 54.98, 54.78, 54.18, 47.59, 47.27, 42.54, 39.91, 39.41, 38.92, 36.07, 35.99, 35.89, 35.70, 35.56, 35.08, 31.47, 30.33, 29.32, 28.12, 27.88, 25.67, 24.26, 23.73, 23.29, 22.71, 22.46, 20.97, 18.57, 16.09, 12.03. C_34_H_62_N_2_O ; MS (ESI) m/z = 515.7 [M+H]^+^

**6β-(1-(2aminoethyl)piperazine)-cholestan-3β-ol 4e:** Purification by column chromatography (silica gel; CH_2_Cl_2_/ MeOH/ NH_4_OH(32%), 7:3:1) afforded a pale yellow solid in 76% yield; – ^1^H NMR (300 MHz, MeOD): δ = 0.64-4.02 (m, 61H) – ^13^C NMR (75 MHz, MeOD): δ = 71.68, 59.08, 58.12, 56.27, 56.06, 54.81, 54.07, 53.91, 53.80, 47.32, 45.91, 45.42, 42.62, 39.94, 39.46, 38.99, 36.12, 35.74, 35.61, 35.16, 31.57, 30.42, 28.17, 27.96, 25.95, 24.33, 23.77, 22.76, 22.52, 21.03, 18.64, 16.25, 12.17. C_33_H_61_N_3_O ; MS (ESI) m/z = 516.6[M+H]^+^

**Procedure for the synthesis of 4g:** A mixture of 6-ketocholestanol (157 mg, 0.39 mmol), titanium(IV) isopropoxide (573 µL, 2.02 mmol) and spermine (202 mg, 1 mmol) in absolute methanol (5 mL) was stirred under argon at room temperature for 12 h. Sodium cyanoborohydride (38 mg, 1 mmol) was then added at -78 ºC and the resulting mixture was stirred for an additional 2 hours. The reaction was then quenched by adding water (1 mL) and stirring was maintained at room temperature for 20 minutes. The resulting inorganic precipitate was filtered off over a pad of Celite and washed with methanol and ethylacetate. The combined organic extracts were dried over Na_2_SO_4_, filtered and concentrated in vacuo to afford the expected crude amino derivative which was purified by flash chromatography affording the expected amino derivative. Purification by column chromatography (silica gel; CH_2_Cl_2_/ MeOH/ NH_4_OH(32%), 7:3:1) afforded a pale yellow oil in 39% yield; this compound can be converted subsequently into its hydrochloride salt as white solid –^1^H NMR (300 MHz, CD_3_OD) : *δ* = 0.65-0.71 (m, 7H), 0.83-0.85 (m, 6H), 0.91-0.93 (m, 3H), 1.09-1.93 (m, 37H), 1.95-2.04 (m, 2H), 2.65-2.81 (m, 2H), 2.90-2.99 (m, 6H), 3.59-3.69 (m, 1H), 4.25 (s, 1H) –^13^C NMR (75 MHz, CD_3_OD) : *δ* = 70.84, 60.25, 56.15, 56.12, 51.96, 48.25, 47.60, 47.49, 46.94, 46.60, 43.56, 42.74, 39.90, 39.40, 39.21, 36.89, 36.22, 35.78, 34.49, 33.33, 32.98, 32.87, 31.15, 29.08, 28.19, 27.14, 26.67, 25.38, 25.28, 24.38, 24.06, 22.66, 21.29, 18.76, 15.94, 12.06. C_37_H_72_N_4_O ; MS (ESI) m/z = 589.5 [M+H]^+^

**Synthesis of 4h and 4i**

The procedure for the synthesis of **4h** and **4i** is like that previously mentioned for **4f-4g** but involving 3-acetoxy-6-ketocholestane and 3-methoxy-6-ketocholestane as starting materials, respectively.

**3β-acetoxy-6β-(spermine)-cholestane 4h:** 41%,  ^1^H NMR (300 MHz, CD_3_OD) : *δ* = 0.58-0.74 (m, 7H), 0.82-0.86 (m, 6H), 0.89-0.93 (m, 3H), 0.96-1.42 (m, 15H), 1.42-1.50 (m, 5H), 1.51-1.57 (m, 6H), 1.58-1.64 (m, 2H), 1.65-1.93 (m, 9H), 1.96-2.64 (m, 12H), 2.65-2.81 (m, 2H), 2.90-2.99 (m, 6H), 4.65-4.74 (m, 1H) –^13^C NMR (75 MHz, CD_3_OD) : *δ* = 170.38, 72.88, 60.25, 56.13, 56.12, 51.96, 49.32, 47.60, 47.49, 46.94, 46.60, 43.56, 42.74, 39.90, 39.40, 39.21, 37.01, 36.22, 35.78, 34.49, 33.33, 32.98, 31.24, 29.08, 28.19, 27.14, 27.10, 26.67, 25.38, 25.28, 24.38, 24.06, 22.63, 21.34, 21.29, 18.76, 15.94, 12.04. C_39_H_74_N_4_O_2_ ; MS (ESI) m/z = 589.5631.5 [M+H]^+^

**3β-methoxy-6β-(spermine)-cholestane 4i:** 71%, ^1^H NMR (300 MHz, CD_3_OD) : *δ* = 0.63-0.73 (m, 7H), 0.82-0.86 (m, 6H), 0.89-0.95 (m, 3H), 0.96-2.04 (m, 39H), 2.43-2.55 (m, 5H), 2.56-2.64 (m, 2H), 2.65-2.81 (m, 2H), 2.90-2.99 (m, 6H), 3.28-3.32 (m, 3H), 3.86-4.07 (m, 1H) –^13^C NMR (75 MHz, CD_3_OD) : *δ* = 77.63, 60.25, 56.15, 56.12, 55.93, 51.96, 49.32, 47.60, 47.49, 46.94, 46.60, 43.56, 42.74, 39.90, 39.40, 39.21, 37.01, 36.22, 35.78, 34.49, 33.33, 32.98, 31.41, 29.08, 28.81, 28.19, 27.14, 26.67, 25.38, 25.28, 24.38, 24.06, 22.64, 21.28, 18.76, 15.94, 12.06.. C_38_H_74_N_4_O ; MS (ESI) m/z = 603.5 [M+H]^+^

**Synthesis of claramine A1**

1. **Synthesis of isopropyl deoxycholate 1**

In a 250 mL two necked round flask was introduced in 70 mL of isopropanol and 30 mL of dichloromethane 10 g of deoxycholic acid (0.0255 mol) and 2.2 g of para-toluene sulfonic acid (0.013 mol). The mixture was heated at reflux under vigorous stirring for 8 hours. The solvents were subsequently removed and 100 mL of dichloromethane was added. The organic phase was washed 3 times with 50mL of NHCO_3_ (10%) solution. The aqueous phases were extracted twice with dichloromethane and the combined organic phases were dried over Na_2_SO_4_, filtered, and concentrated in vacuo to afford the expected product as a white solid in 90% yield. NMR ^1^H (250 MHz, CDCl_3_) : δ (ppm) = 4.94 (m, 1H), 3.92 (m, 1H), 3.53 (m, 1H), 2.31-2.09 (m, 2H), 1.78-0.85 (m, 38H), 0.61 (s, 3H). NMR ^13^C (63 MHz, CDCl_3_) : δ (ppm) = 173.68, 72.86, 71.37, 67.17, 47.99, 47.05, 46.32, 41.95, 36.23, 35.86, 35.17, 35.11, 33.97, 33.38, 31.61, 30.80, 30.20, 28.49, 27.42, 27.05, 26.01, 23.58, 22.99, 21.70, 17.06, 12.53.

1. **Synthesis of 3-oxo isopropyl deoxycholate 2**

In a 250 mL two necked round flask was introduced in 100 mL of toluene and 50 mL of acetone 11 g of isopropyl deoxycholate **1** (0.025 mol). 2 equivalents of aluminum *tert*-butoxide (12.3 g, 0.050 mol) were subsequently added and stirring was maintained under reflux for 12 hours. 50 mL of a 2N H_2_SO_4_ solution was added and the mixture was stirred for an additional 1 hour.

The organic phase was washed 3 times of a 2N H_2_SO_4_ solution and 50 mL of water. The combined organic phases were dried over Na_2_SO_4_, filtered, and concentrated in vacuo to afford a crude product which was purified by flash chromatography on silica gel (ethylacetate/ petroleum ether (1/1)). The expected 3-oxo isopropyl deoxycholate **2** was successfully obtained as a white solid in 70% yield. NMR ^1^H (250 MHz, CDCl_3_) : δ (ppm) = 5.00 (m, 1H), 4.04 (m, 1H), 2.46-2.12 (m, 4H), 2.06-0.96 (m, 35H), 0.71 (s, 3H). NMR ^13^C (63 MHz, CDCl_3_) : δ (ppm) = 212.74, 173.57, 72.98, 67.34, 48.22, 47.59, 46.70, 44.30, 42.33, 37.04, 36.92, 35.87, 35.05, 34.45, 34.11, 31.76, 31.00, 29.06, 27.40, 26.64, 25.55, 23.58, 22.38, 21.81, 17.42, 12.75.

1. **Synthesis of 3-spermino isopropyl deoxycholate (claramine A1)**

In a 100 mL two necked round flask was introduced 100 mL of methanol. A mixture of the ketone **2** (3.5 g, 8 10^-3^mol), titanium(IV) isopropoxide (7.15 mL, 2.4 10^-2^ mol), and spermine (3.2g, 1.6 10^-2^ mol) was stirred under argon at room temperature for 24 hours. After cooling the flask at -20°C, sodium borohydride (0.9 g, 2.4 10^-2^ mol) was then added and the resulting mixture was stirred for additional 12 hours. The reaction was then quenched by adding water (4 mL). Stirring was continued at room temperature for 1 hour then the reaction mixture was filtered over a pad of Celite which was subsequently rinsed with NH_4_OH and methanol. The mixture was concentrated in vacuo to afford the expected crude compound which was purified by flash chromatography on silica gel using CH_2_Cl_2_/MeOH/NH_4_OH (32%) 7:3:1 as eluent. The expected claramine A1 was obtained as a viscous yellow oil in 52% yield. NMR ^1^H (250 MHz, CD_3_OD) : δ (ppm) = 4.96 (m, 1H), 3.94 (m, 1H), 2.88-2.46 (m, 13H), 2.39-2.18 (m, 2H), 2.04-1.12 (m, 40H), 1.01-0.95 (m, 7H), 0.71 (s, 3H). NMR ^13^C (63 MHz, CD_3_OD) : δ (ppm) = 175.66, 74.15, 68.95, 59.06, 50.74, 50.70, 49.30, 48.97, 48.42, 48.27, 47.77, 45.83, 44.08, 40.78, 37.58, 37.14, 36.82, 35.97, 35.92, 34.94, 34.59, 33.56, 32.72, 32.45, 30.52, 29.98, 28.84, 28.65, 28.40, 28.29, 27.64, 25.04, 24.10, 22.27, 17.71, 13.37. MS (ESI^+^): m/z 619.5519 ([M+H]^+^)

**Synthesis of soluble derivatives 4a-4i as their hydrochloride salts**

General procedure illustrated for Claramine A1. Claramine A1 is dissolved in a minimum of anhydrous methanol and anhydrous HCl solution in diethylether (2M, 8 equivalents) was slowly added under vigorous. The formed precipitate was filtrated, washed with anhydrous diethylether and dried under vacuum. The claramine A1 hydrochloride salt is obtained in a quantitative yield as a white solid stable to air and moisture.
